# Allergic manifestations in inborn errors of immunity: a systematic scoping review

**DOI:** 10.3389/fimmu.2025.1666600

**Published:** 2025-10-10

**Authors:** Lars Marbet, Esther Gujer, Marlene H. Münger, Michael P. Killian, Andrea A. Mauracher, Maarja Soomann, Johannes Trück, Jana Pachlopnik Schmid

**Affiliations:** ^1^ Division of Immunology and Children’s Research Center, University Children’s Hospital Zurich, Zurich, Switzerland; ^2^ Division of Allergy and Immunology, Department of Pediatrics, Children’s Hospital of Philadelphia, Philadelphia, PA, United States; ^3^ Division of Allergy and Children’s Research Center, University Children’s Hospital Zurich, Zurich, Switzerland; ^4^ Pediatric Immunology, Medical Faculty, University of Zurich, Zurich, Switzerland

**Keywords:** inborn errors of immunity, primary immunodeficiency, allergy, atopy, immune dysregulation, systematic scoping review

## Abstract

**Background:**

Allergic diseases resulting from aberrant immune function are frequently observed in patients with inborn errors of immunity (IEI). However, the underlying monogenic disorders may not be initially diagnosed, which leads to delays in appropriate diagnosis and treatment. Although recent studies have highlighted the link between IEI and allergic conditions, earlier case series might not have been fully exploited.

**Objective:**

To compile a comprehensive database of IEI cases in the literature, analyze the prevalence and characteristics of allergic diseases, and assess the latter’s association with other forms of immune dysregulation.

**Method:**

A systematic scoping review of the literature.

**Results:**

A total of 738 articles (reporting on 3050 individual patients) were included. 226 (7.4%) of the patients were described as suffering from an allergy. Monogenetic diseases associated with a marked prevalence of allergy were found in various IEI subgroups. Food allergy was most frequently reported (n=172, 76.1%), followed by allergic rhinitis (n=56, 24.8%). The presence of allergy in patients with IEI was generally associated with the absence of other forms of immune dysregulation, although there were notable exceptions. The overall prevalence of atopic conditions (food allergy, allergic rhinitis, asthma, and eczema) in the dataset was 26.9% (n=821).

**Conclusion:**

This systematic scoping review emphasized the relevance of allergic diseases as a manifestation of immune dysregulation in IEI. Our findings might raise awareness of allergy in IEI among clinicians and researchers and constitute a valuable resource for better diagnosis and management of these conditions.

## 1 Introduction

Allergic diseases and atopy are hallmark features of certain inborn errors of immunity (IEI) and sometimes even constitute the primary manifestation of these conditions (1–4). Although IEI were initially termed “primary immunodeficiencies” [due to their association with increased susceptibility to infection (5)], the term now encompasses a broader spectrum of immune dysregulation disorders, including autoimmunity, autoinflammation, allergy, and malignancy (1, 6). Mechanistic studies of atopy in IEI have identified changes in cytokine signaling, impairments in T-cell receptor signaling, and intrinsic mast cell dysfunction (3). To better classify the monogenic disorders associated with atopy and emphasize the extension of the allergic phenotype beyond susceptibility to infection, Lyons and Milner suggested the term “primary atopic disorder” (7). Similarly, the term “primary immune regulatory disorder” (PIRD) underscores the immune dysregulation that is characteristic of IEI (8). At least 485 distinct IEIs were listed in a classification published in 2022 (6), and that number has now risen to 508 (9). Although each IEI remains rare, the estimated combined prevalence ranges from 1:1,000 to 1:5,000 (10). The phenotypic heterogenicity and rarity of IEI are compounded by limited awareness among clinicians; this often results in delayed diagnosis and treatment and thus high morbidity and mortality rates (11). The same may also be true for primary atopic disorders, which may initially present solely as atopic manifestations and can thus be misdiagnosed as common allergic diseases (2, 12). Recognizing IEI in the context of severe allergic phenotypes is crucial (3) because their progression and management differ significantly from those associated with non-IEI allergic conditions (13).

Atopic disorders include asthma, atopic eczema, allergic rhinitis, and food allergy and constitute a growing public health challenge. Globally, asthma affects more than 300 million individuals (14), while atopic eczema affects 15-20% of children and 1-3% of adults (15, 16). Allergic rhinitis affects up to 25% of the population (17), and the prevalence of food allergy can be as high as 10% (18). These conditions impose substantial social, psychological and financial burdens on patients and their families (17, 19–21) and significantly reduce quality of life (22, 23). Severe allergic reactions (including fatal anaphylaxis) remain a critical concern – particularly with food allergens like peanuts and tree nuts (24). Rising prevalence rates since the 1980s – including an increase in adult-onset allergies (21, 25, 26) – have prompted efforts to understand the etiology of atopic disorders. Factors such as genetic predispositions, microbiome alterations, lifestyle changes, environmental influences, and epithelial barrier dysfunction have all been implicated (27, 28). The dual-exposure hypothesis of food allergy highlights enteral and cutaneous exposures to allergens as drivers (29–31), and emerging evidence suggests that lung exposure via air pollution also has a role (25). Climate change and regional effects may further influence the incidence of allergy (32, 33).

The study of monogenic disorders with allergic phenotypes provides unique insights into the pathogenesis of allergic diseases and offers potential avenues for novel treatments of both syndromic and non-syndromic allergic conditions (7). Although allergy has been explored in specific IEI or subsets of IEI (34–36), comprehensive investigations of allergic manifestations across a broad spectrum of IEI are still required.

In 2021, we analyzed immune dysregulation patterns in IEI by using the GARFIELD mnemonic (“granuloma”, “autoimmunity”, “recurrent fever”, “eczema”, “lymphoproliferation”, and “intestinal disease”) to encompass key features (37, 38). The field has evolved rapidly since then: novel IEI are increasingly identified in patients presenting primarily with severe allergic phenotypes, while allergic manifestations in previously described IEI are also being recognized more readily. To capture this emerging evidence, we have used advanced search strategies to expand our database. This effort has resulted in (i) an updated manuscript on immune dysregulation patterns in IEI (in preparation) and (ii) the present systematic scoping review of literature data on the association between IEI and allergy, with a particular focus on allergic manifestations of previously described IEI and the concomitant occurrence of allergic diseases and other features of immune dysregulation.

## 2 Methods

This scoping review was conducted in alignment with the PRISMA-ScR (Preferred Reporting Items for Systematic Reviews and Meta-Analyses extension for Scoping Reviews) guidelines (39). No formal protocol was registered for this scoping review.

### 2.1 Literature search

We searched the Scopus, EMBASE, PubMed and Ovid databases for reports published between January 1^st^, 2000, and February 4^th^, 2021. The start date of 2000 was chosen because that was the year in which the International Human Genome Sequencing Consortium announced the “completion of a working draft of the human genome sequence” (40). The search strategy included the keywords “primary immune regulatory disorders”, “inborn errors of immunity”, “primary immunodeficiency disorders”, “primary immunodeficiency”, “genetic Immunodeficiency” combined with “granuloma”, “autoimmunity”, “autoimmune”, “autoinflammation”, “recurring fever”, “periodic fever”, “chronic inflammation”, “eczema”, “rash”, “lymphoproliferation”, “lymphadenopathy”, “splenomegaly”, “hepatosplenomegaly”, “inflammatory bowel disease”, “enteropathy”, “chronic diarrhea”, “intestinal disease”, and “allergy”, together with a list of all IEI genes given by the International Union of Immunological Societies (IUIS) in its 2019 report (10). The term “human immunodeficiency virus” was excluded. The full search terms are given in the **Supplementary Material**.

### 2.2 Web application

A custom web application was built and used to screen publications, assess eligibility, and extract data. Access to the application’s webpage can be granted on request.

### 2.3 Eligibility criteria

Eligible reports had to be published in English and accessible through the libraries at the University of Zurich or Swiss Federal Institute of Technology in Zurich. Only original research manuscripts from peer-reviewed journals were considered. Accepted study designs included randomized controlled trials, case-control studies, case reports, cohort studies, conference papers, and letters to the editor. The study populations consisted of patients presenting with granuloma, autoimmunity, recurrent fever, eczema, lymphoproliferation, gastrointestinal disease, or allergy and who also had a genetically confirmed IEI, as defined in the IUIS 2019 report. We excluded narrative reviews, systematic reviews, editorials, commentaries, unpublished manuscripts, dissertations, government reports, books and book chapters, conference proceedings, guidelines, consensus statements, cross-sectional studies, qualitative studies, non-systematic reviews, studies without reported methods, and cadaveric, biomechanical, or laboratory studies. Generally, earlier publications tended not to mention distinct genomic variants, which is why records published before 2000 were not considered for further screening. When a given patient was described in several publications, only the first report was included and the subsequent reports were excluded.

### 2.4 Screening and data extraction

Using the custom web application, two reviewers independently screened publication titles and abstracts from the database search results, removing records that clearly did not meet eligibility criteria. Disagreements were resolved by a third reviewer. Full-text articles of the remaining records were then assessed for eligibility by two reviewers. 186 publications that had already been included in our first review on immune dysregulation patterns in IEI (based on a search from the inception of the databases until May 8^th^, 2017) met all the eligibility criteria and did not need to be screened a second time (37). Data extraction was performed within the same custom-built web application, with the workload divided among six reviewers. Any uncertainties were re-evaluated by an additional reviewer and resolved through discussion and consensus. For each patient, clinical symptoms were extracted and recorded according to the Human Phenotype Ontology (HPO, https://hpo.jax.org/app/, version July 2022) classification system. When phenotypes were described in terms not directly aligned with the HPO terminology, the reviewers agreed on appropriate transcription into HPO terms, and these decisions were documented. Similarly, when recurrent fever was attributed by the authors to infection, the HPO term “fever” was used, while “recurrent fever” was reserved for cases with an unspecified or explicitly autoinflammatory etiology. Specific diagnoses lacking a corresponding HPO code (e.g., multiple sclerosis) were recorded in a designated free-text field to preserve all available information.

Monogenetic disorders were annotated using the 2019 IUIS list, with documentation of the relevant gene and mode of inheritance. Pathogenic mutations were recorded on the DNA and/or protein levels. Additional patient characteristics such as sex, age at symptom onset, and country of origin were also documented. If the country of origin was not reported, the nationality of the first author was used as a proxy.

For the purposes of this review, all patients with recorded instances of allergy (explicitly reported by the authors as “allergy” or described with terms indicative of allergic conditions such as “allergic asthma”) were reassessed. This process involved re-examining the original articles to survey and, if necessary, refine the documentation of allergies.

### 2.5 Data analysis

In this systematic scoping review, we extracted patient-level data and excluded individuals who did not meet the search term criteria prior to data analysis with R (41) using RStudio (42). Specifically, 207 cases without any HPO terms assigned to immune dysregulation were discarded, along with four patients who had asthma as the sole form of immune dysregulation (as asthma was not included in the original keyword search). Eight additional cases with genetic disorders not included in the 2019 IUIS classification (10) or its 2022 update (6) were excluded, while three patients with SOCS1 haploinsufficiency were included (given its introduction in the IUIS 2022 update). Eleven patients with somatic mutations only were also excluded. Allergy-related HPO terms identified during data extraction were categorized into five groups: food allergy, allergic rhinitis, drug allergy, other, or (when specific details were unavailable) unknown. Likewise, HPO terms related to other forms of immune dysregulation were consolidated into broader categories to facilitate analysis. For example, “lymphoproliferative disorder”, “lymphadenopathy”, “hepatomegaly”, and “splenomegaly” were grouped under the category “lymphoproliferation”. Similarly, psoriasis was combined with other inflammatory skin diseases under “rash”. The definitions of allergy categories and forms of immune dysregulation can be found in the **Supplementary Material**.

**Figure 1:**
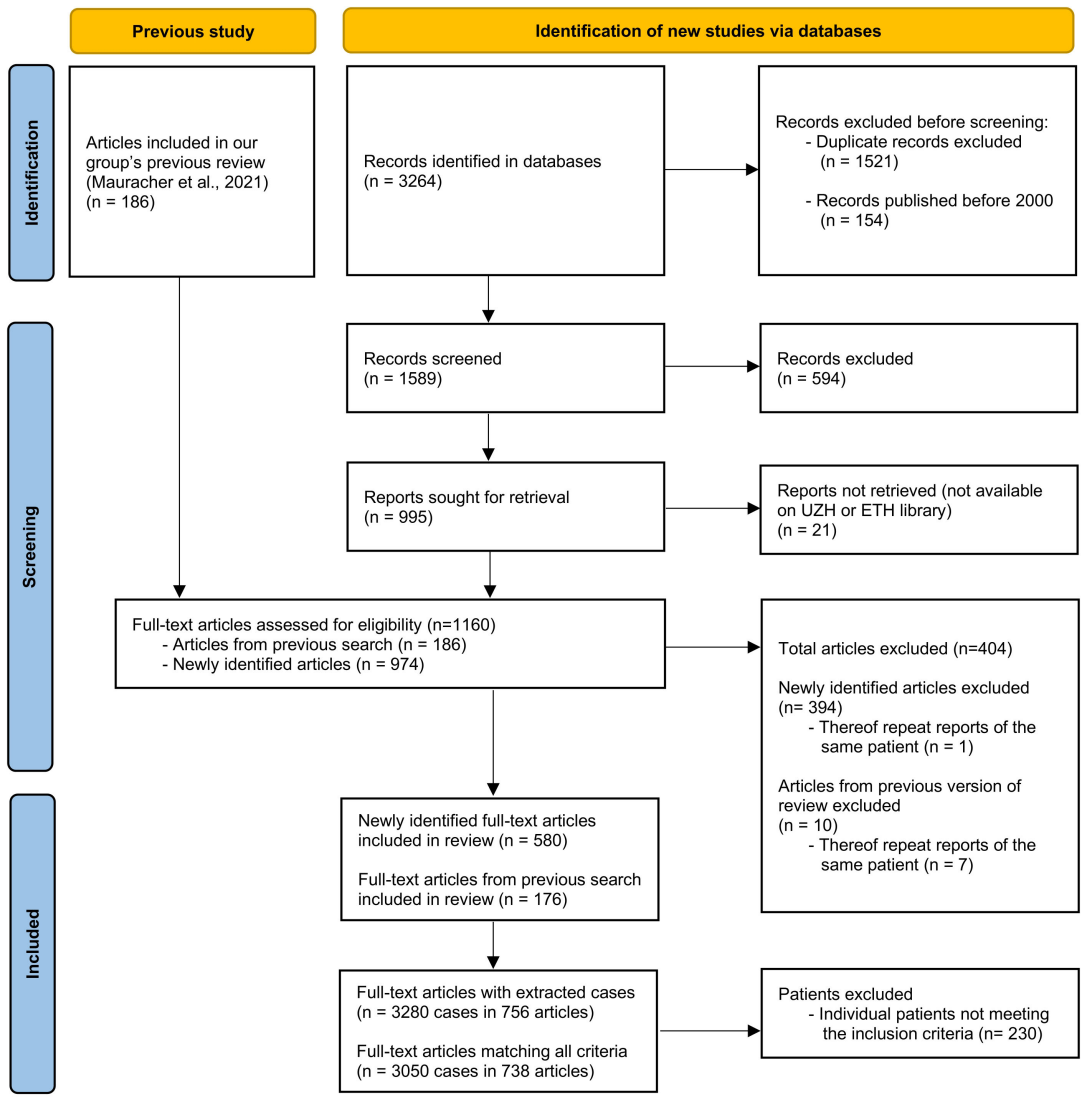
Flowchart according to the Preferred Reporting Items for Systematic Reviews and Meta-Analyses (PRISMA) statement (44), depicting the number of original articles identified and screened and included or excluded.

Scatter plots, bubble plots, and heatmaps were created for data visualization (43). The flowchart presented in **Figure 1** was created in accordance with the PRISMA guidelines (44). Data were summarized at two levels: (i) individual genes, and (ii) subgroups based on the main and sub-tables from the IUIS classification (6, 10) (see **Supplementary Table S1**). Heatmaps were restricted to gene-disease groups with sufficient case numbers to allow for meaningful interpretation. Gene names appearing in figures are followed by the disease mechanism (e.g., *CARD11* (AD DN)) in some instances where it was of relevance.

## 3 Results

### 3.1 Data characteristics

After the removal of duplicates and records published before 2000, the search identified 1589 records (see **Figure 1**). After screening and selection, 580 articles were retained for data extraction; none of these had been included in our previous review (37). Of the 186 articles included in the previous review, 176 were included in the present review with extraction of patient data. The most common reasons for exclusion were incomplete documentation of genetic mutations and inability to identify individual patients or match clinical information to specific individuals. Ultimately, 756 publications describing 3280 patients were included in the present review. Of these, 3050 patients (from 738 publications) met all eligibility criteria and were included in the final analysis.

### 3.2 Involved genes

Of the 3050 patients included in the analysis, 226 (7.4%) were reported to have at least one allergy and are referred to henceforth as “allergy patients”. When cases only included due to the presence of allergy were deducted, the allergy prevalence amounted to 6.9% of the remaining n=3033 patients. The IEI genes most frequently implicated in the allergy patients were Dedicator of cytokinesis 8 (*DOCK8*, 51.2%, n=107), autosomal dominant negative Caspase recruitment domain family member 11 (*CARD11* (AD DN), 7.7%, n=16), and then Forkhead box P3 (*FOXP3*, 5.3%, n=11), Phospholipase C gamma 2 (*PLCG2*, 5.3%, n=11) and X-linked Wiskott-Aldrich syndrome (*WAS* (XL), 5.3%, n=11). Another 12 IEI genes were reported in 2 to 9 allergy patients each. For 17 genes, only one allergy patient was described, and no allergy cases were reported for 164 of the 198 genes in the dataset. Full results on the gene and subgroup levels are given in **Supplementary Table S2**.

**Figure 2:**
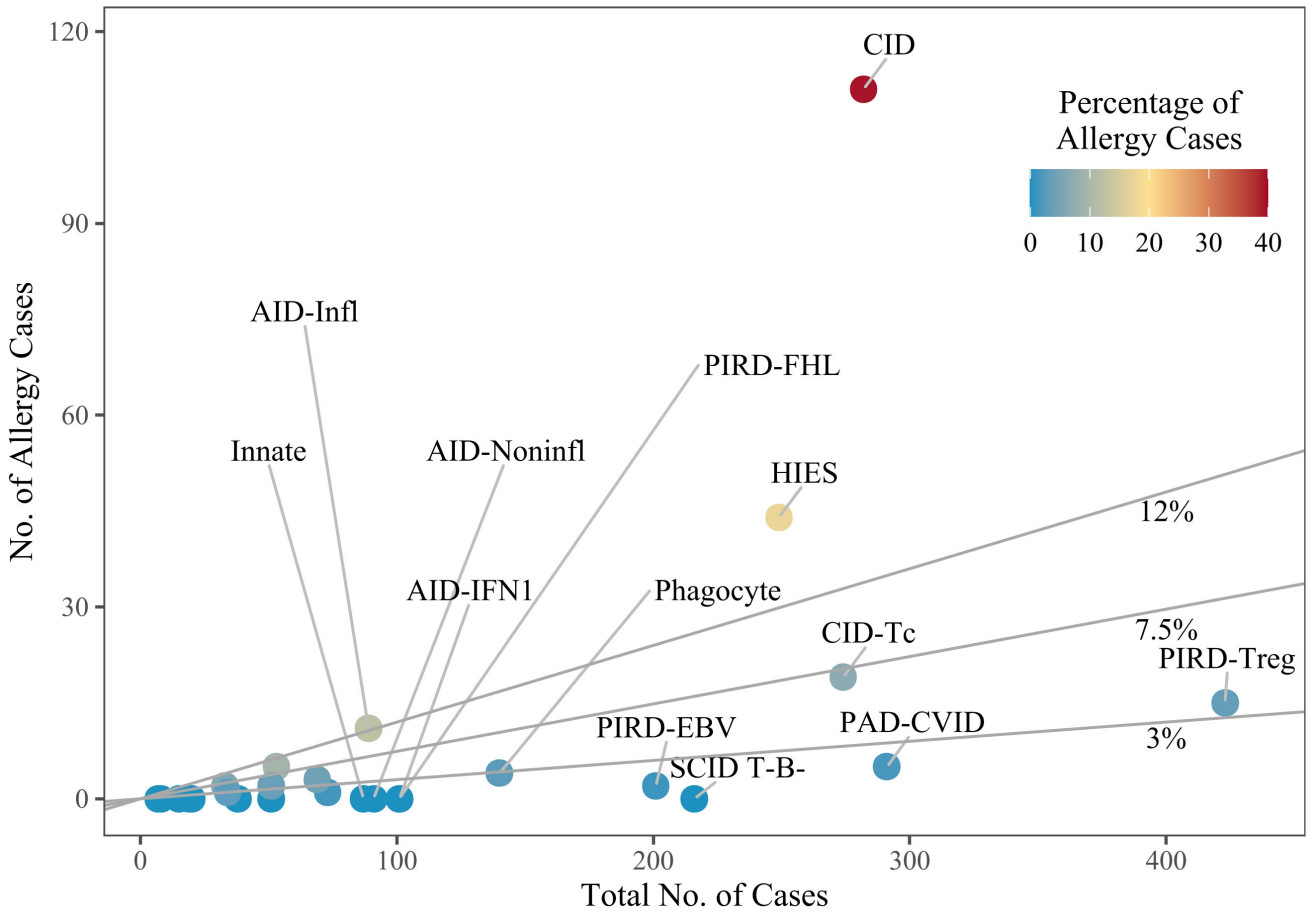
Subgroups, by the number of cases. Dots represent individual subgroups and are colored according to the percentage of allergy cases; a line marks the overall mean percentage of 7.5%, and lines for 3% and 12% are given for visual guidance. Subgroups with only few cases overall are not labelled. The definitions of the subgroups can be found in **Supplementary Table S1**. SCID, severe combined immunodeficiency; CID, combined immunodeficiency (less profound than SCID); CID-Tc, immunodeficiency with congenital thrombocytopenia; HIES, hyper IgE syndromes; PAD-CVID, predominantly antibody deficiencies, common variable immunodeficiency (CVID) phenotype; PIRD-FHL, familial hemophagocytic lymphohistiocytosis; PIRD-Treg, regulatory T cell defects; PIRD-EBV, susceptibility to Epstein-Barr virus and lymphoproliferative conditions; Phagocyte, phagocyte defects; Innate, intrinsic/innate immunity defects; AID-IFN1, type 1 interferonopathies; AID-Infl, inflammasome defects; AID-Noninfl, non-inflammasome related conditions.

When examining the proportion of allergy patients with variants in a given gene (for genes with at least 20 cases), *DOCK8* had the highest proportion (66.1% of n=162), followed by *CARD11* (AD DN) (53.3% of n=30), Actin-related protein 2/3 complex subunit 1B (*ARPC1B*, 38.1% of n=21), and *PLCG2* (37.9% of n=29) (**Figure 2**). In contrast, when considering genes with at least 100 cases, those such as Lipopolysaccharide-responsive beige-like anchor (*LRBA*, 0% of n=176) and Recombination activating gene 1 (*RAG1*, 0% of n=141) had no reported allergy patients, while TNF receptor superfamily member 13B (*TNFRSF13B*) had only one allergy patient (1% of n=104).

We compared the entries from the IUIS tables (6) that mentioned allergy or atopy as associated features with the genetic disorders identified in our analysis as having a high proportion of allergy patients (**Table 1**).

**Table 1:** Entries from the IUIS tables in Tangye et al. (6) with a mention of allergy or atopy as an associated feature, as well as genetic variants with no mentioned association with atopy but which exhibited an allergy prevalence of over 35% in our analysis.

Information from the IUIS tables				Study results			
Disease	Gene implicated	Inheritance	Associated features	Total number of cases	Number of allergy cases	Proportion	Comment
DOCK8 deficiency	*DOCK8*	AR	Severe atopy/allergic disease	162	107	66%	
IKAROS GOF	*IKZF1*	AD GOF	Allergy	–	–	–	Newly introduced in the IUIS 2022 update
Loeys-Dietz syndrome	*TGFBR1/* *TGFBR2*	AD	Food allergies	5	5	100%	
Comel-Netherton syndrome	*SPINK5*	AR	Atopic diathesis	3	2	67%	
PGM3 deficiency	*PGM3*	AR	Severe atopy	18	9	50%	
CARD11 deficiency (heterozygous DN)	*CARD11*	AD LOF	Variable atopy, food allergies	30	16	53%	
RLTPR deficiency	*CARMIL2*	AR	Atopy	14	1	7%	
NCKAP1L deficiency	*NCKAP1L*	AR	Atopy	–	–	–	Newly introduced in the IUIS 2022 update
Arp2/3-mediated filament branching defect	*ARPC1B*	AR	–	21	8	38%	A relatively high proportion of allergy patients, although allergy was not an “associated feature”
IL6 signal transducer deficiency	*IL6ST*	AR	–	10	6	60%	
PLAID	*PLCG2*	AD GOF	–	29	11	38%	

AR, autosomal recessive; AD, autosomal dominant; GOF, gain-of-function; LOF, loss-of-function.

### 3.3 Allergy categories

Food allergies were the most common category (affecting 76.1% (n=172) of the allergy patients), followed by allergic rhinitis (24.8%, n=56) and drug allergies (6.6%, n=15). There was one case of insect allergy and one case of latex allergy. Furthermore, 15.0% (n=34) of patients had unspecified allergies.

**Figure 3:**
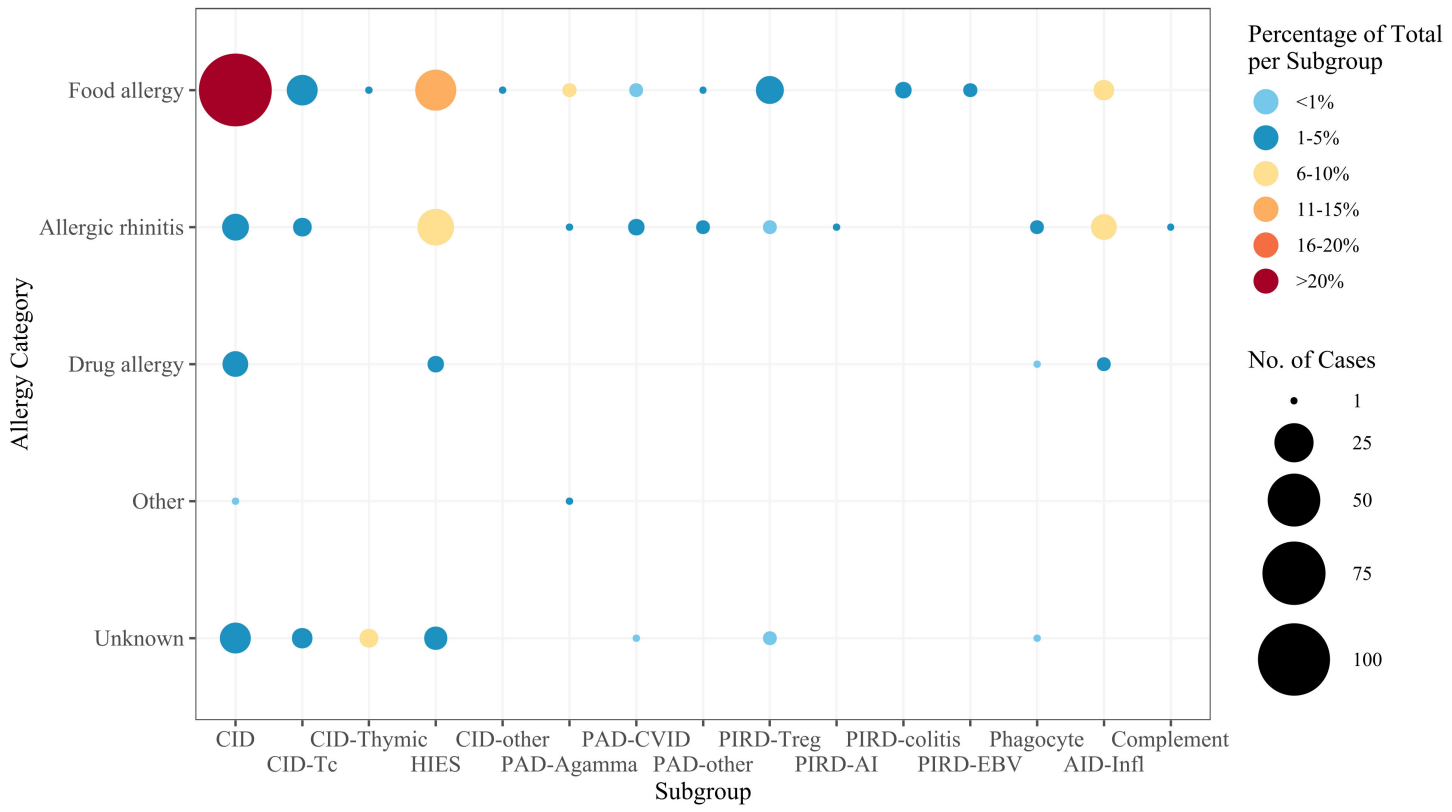
Frequency of allergies in IEI subgroups. The size of the dot is proportional to the absolute number of allergy cases, and the color scale represents the frequency of each allergy category among all patients within the specific subgroup. For the subgroup definitions, see **Supplementary Table S1**. CID, combined immunodeficiency (less profound than SCID); CID-Tc, immunodeficiency with congenital thrombocytopenia; CID-Thymic, thymic defects; HIES, hyper IgE syndromes; CID-other, other defects of CID with associated features; PAD-Agamma, agammaglobulinemia; PAD-CVID, predominantly antibody deficiencies, CVID phenotype; PAD-other, other predominantly antibody deficiencies; PIRD-Treg, regulatory T cell defects; PIRD-AI, autoimmunity with or without lymphoproliferation; PIRD-colitis, immune dysregulation with colitis; PIRD-EBV, susceptibility to Epstein-Barr virus and lymphoproliferative conditions; Phagocyte, phagocyte defects; AID-Infl, inflammasome defects; Complement, complement deficiencies.

When grouping allergy cases by IEI subgroup and considering only those with at least 10 cases, the highest prevalence of food allergy was observed in the “Combined immunodeficiency (CID), generally less profound than severe combined immunodeficiency (SCID)” subgroup (91.9%, n=102) (**Figure 3**). Allergic rhinitis was most prevalent in the “inflammasome defects” subgroup (81.8%, n=9), and patients with unspecific allergies were found particularly in the “immunodeficiency with congenital thrombocytopenia” subgroup (26.3%, n=5). Notably, the single cases of latex and insect allergy were reported in a patient with DOCK8 deficiency and a patient with Bruton’s tyrosine kinase (BTK) deficiency, respectively.

821 of the 3050 patients (26.9%) had at least one atopic condition. After the exclusion of cases of eczema not explicitly labelled as “atopic”, the prevalence dropped to 10.8% (n=328). Food allergy was reported in 5.6% of patients overall, with the highest prevalence in those with DOCK8 deficiency. Asthma was noted in 3.7%, with a high prevalence in patients with CARD11 (AD DN) or Interleukin 6 cytokine family signal transducer (IL6ST) deficiency. Allergic rhinitis was reported in 1.8% of patients (mostly those with PLCG2 deficiency). Atopic dermatitis/eczema affected 24.6%, and 4.2% were explicitly labeled as atopic dermatitis (mostly among patients with Phosphoglucomutase 3 (PGM3) deficiency). For patients with DOCK8 deficiency, food allergy was reported in 61.7%, asthma in 22.2%, allergic rhinitis in 4.9%, and eczema in 89.5% (15.4% specifically atopic eczema). In CARD11 (AD DN) deficiency, food allergy was reported in 33.3%, asthma in 50%, allergic rhinitis in 23.3%, and eczema in 86.7% (80% specifically atopic eczema). The prevalence of atopic manifestations is described in detail in **Supplementary Table S2**.

### 3.4 Associations with immune dysregulation

To explore potential associations between allergy and other forms of immune dysregulation, proportions were calculated for two scenarios: (i) the proportion of allergy patients with another type of immune dysregulation, and (ii) the proportion of patients with a specific type of immune dysregulation who also had an allergy.

**Figure 4:**
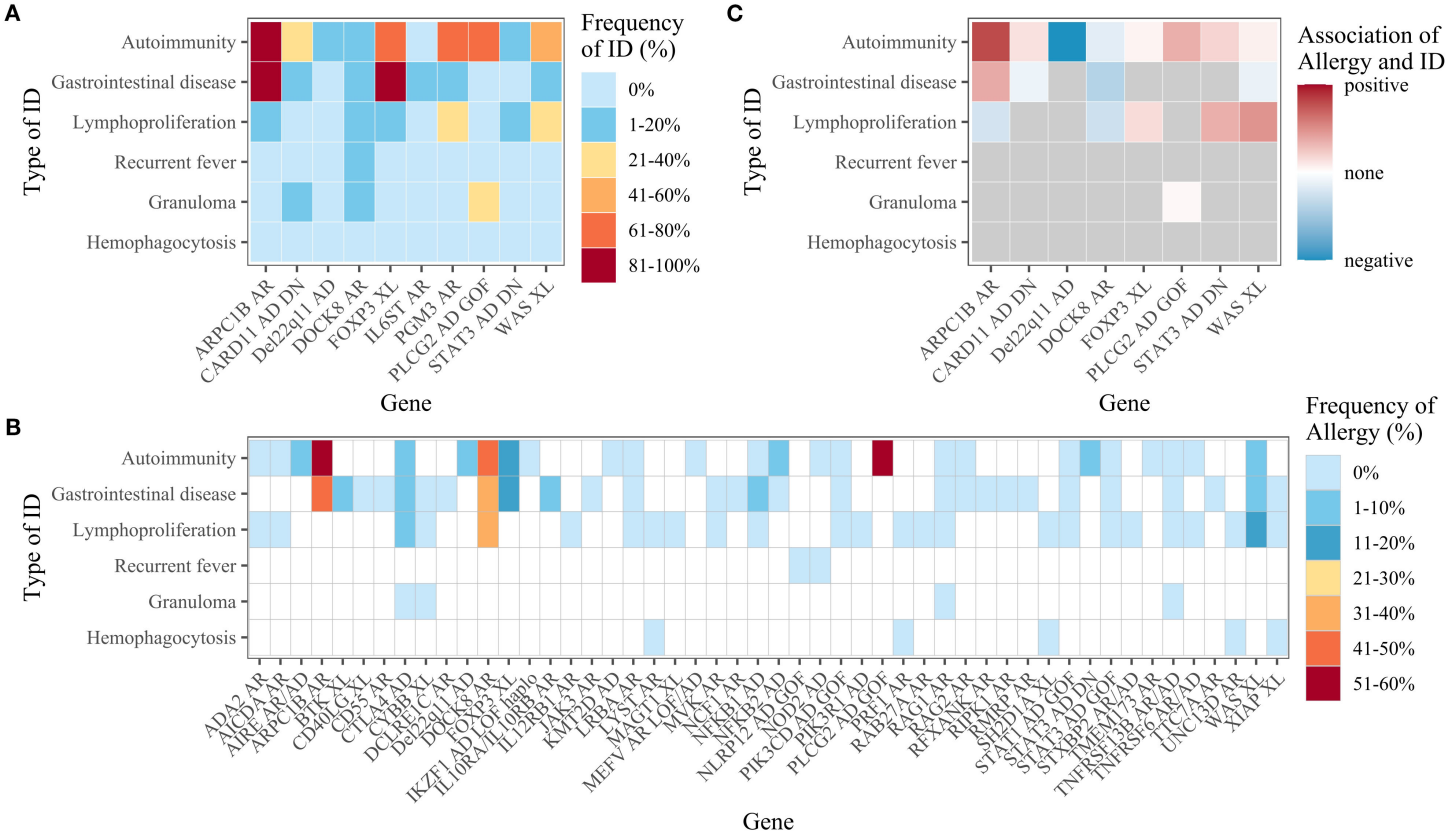
**(A)** Frequency of immune dysregulation (ID) among patients with allergy, by gene. Only genes with at least five allergy patients are shown. **(B)** Frequency of allergy among patients with immune dysregulation, by gene. Data are only shown if there were at least 10 patients with the immune dysregulation in question. Genes with less than 10 patients for every type of immune dysregulation are not displayed. **(C)** The association between allergy and immune dysregulation, by gene. Only genes with at least five allergy and non-allergy patients were considered. The degree of association was only shown if there were at least five patients with the stated type of immune dysregulation and five patients without.

**Figure 5:**
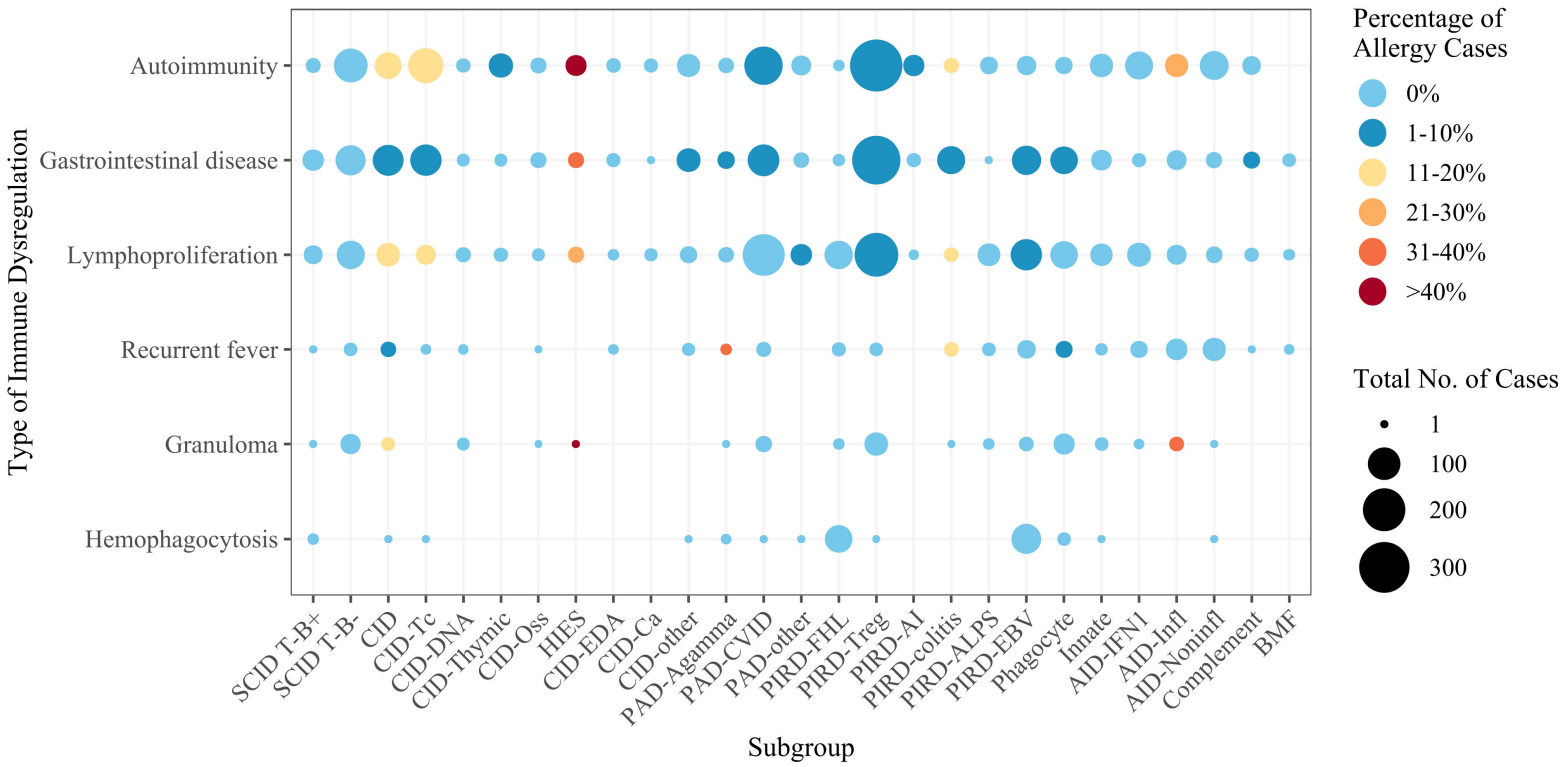
Numbers of cases by IEI subgroup and by type of immune dysregulation. The color scale reflects the percentage of allergy cases. For the subgroup definitions, see **Supplementary Table S1**. SCID, severe combined immunodeficiency; CID, combined immunodeficiency (less profound than SCID); CID-Tc, immunodeficiency with congenital thrombocytopenia; CID-DNA, DNA repair defects; CID-Thymic, thymic defects; CID-Oss, immuno-osseous dysplasia; HIES, hyper IgE syndromes; CID-EDA, ectodermal dysplasia; CID-Ca, calcium channel defects; CID-other, other defects of CID with associated features; PAD-Agamma, agammaglobulinemia; PAD-CVID, predominantly antibody deficiencies, CVID phenotype; PAD-other, other predominantly antibody deficiencies; PIRD-FHL, familial hemophagocytic lymphohistiocytosis; PIRD-Treg, regulatory T cell defects; PIRD-AI, autoimmunity with or without lymphoproliferation; PIRD-colitis, immune dysregulation with colitis; PIRD-ALPS, autoimmune lymphoproliferative syndrome; PIRD-EBV, susceptibility to Epstein-Barr virus and lymphoproliferative conditions; Phagocyte, phagocyte defects; Innate, intrinsic/innate immunity defects; AID-IFN1, type 1 interferonopathies; AID-Infl, inflammasome defects; AID-Noninfl, non-inflammasome related conditions; Complement, complement deficiencies; BMF, bone marrow failure.

For the dataset as a whole, the proportion of allergy patients also suffering from another type of immune dysregulation ranged from 1.8% (for recurrent fever) to 25.7% (for autoimmunity). The frequency of patients with immune dysregulation suffering from allergy ranged from 1.7% (for lymphoproliferation) to 4.5% (for autoimmunity). There were no cases of concomitant allergy and hemophagocytosis. On both the gene level (**Figure 4A**) and the IEI subgroup level (**Supplementary Figure S1**), the types of immune dysregulation most frequent in allergy patients were autoimmunity and gastrointestinal disease. Conversely, allergy was most frequent in patients with autoimmunity or gastrointestinal disease on both the gene level (**Figure 4B**) and the IEI subgroup level (**Supplementary Figure S2**). Irrespective of allergy frequency, these two were the most frequent types of immune dysregulation for most subgroups (**Figure 5**). Within the inflammasome subgroup (AID-Infl), allergy and recurrent fever did not co-occur. All 11 allergy cases were due to PLCG2 deficiency leading to PLAID (PLCG2 associated antibody deficiency and immune dysregulation), whereas the 31 cases of recurrent fever were observed in other conditions, most notably Familial cold autoinflammatory syndrome 2 caused by *NLRP12* variants and Familial Mediterranean fever caused by *MEFV* variants.

The frequency of immune dysregulation in the presence of allergy and the frequency of allergy in the presence of immune dysregulation were compared, and the putative association between allergy and immune dysregulation was expressed for each gene as an odds ratio. For the dataset as a whole, there were negative relationships between allergy and all forms of immune dysregulation, while some distinct positive relationships existed on both the gene level (**Figure 4C**) and the IEI subgroup level (**Supplementary Figure S3**).

## 4 Discussion

Our systematic scoping review of literature data on IEI patients with immune dysregulation included 738 studies and 3050 patients, 226 (7.4%) of whom had an allergy. The highest prevalence of allergy was found in patients suffering from genetic disorders caused by variants in *DOCK8*, *CARD11* (AD DN), *ARPC1B*, and *PLCG2*, while there were no occurrences of allergy in patients with defects in the *LRBA* and *RAG1* genes. Food allergies were the most common kind of allergy. Allergy patients were less frequently affected by other forms of immune dysregulation, although there were exceptions to this rule. Our data provide insights into the prevalence and relevance of allergy in the setting of IEI and reveal associations with other diseases.

The proportion of IEI patients with allergy in the present review (7.4%) was higher than in our previous review on immune dysregulation in IEI (4%) (37). Overall, our findings concerning allergy as a typical feature of IEI were in line with the literature data. For instance, allergy is known to be frequent in people with a CARD11 (AD DN) deficiency (45, 46), a DOCK8 deficiency (4, 46) or a PGM3 deficiency (7, 47). While the IUIS 2022 classification report (6) states that allergy or atopy is characteristic for these conditions, it does not do so for ARPC1B, PLCG2, and IL6ST deficiencies – all of which had a high prevalence of allergy in our study. However, other researchers have suggested that allergic conditions are a common presentation of these IEI (13, 46, 48, 49).

Many IEI stood out because of a lack of reported allergy among affected patients. For RAG SCID, this lack can be explained by the absence of IgE or type 2 T helper (Th2) cells (3). However, people with a hypomorphic loss of function variant can present with Omenn syndrome and concomitant eczema and elevated IgE levels but with no known allergen specificity (48). To our surprise, there were two reports of allergy in patients with BTK deficiency (also known as X-linked agammaglobulinemia); this disease is characterized by B cell deficiency, the absence of plasma cells, and very low serum levels of all immunoglobulin isotypes (50). In a report on the prevalence of food allergy in IEI patients within the United States Immunodeficiency Network (USIDNET), there was also one case of food allergy among the 332 patients with X-linked agammaglobulinemia (51). For familial Mediterranean fever, Celiksoy et al. found the prevalences of allergic rhinitis, food allergy and atopic dermatitis in their cohort of patients with *MEFV* variants to be 75%, 7.5% and 22%, respectively (52). However, none of our 21 patients reportedly exhibited any manifestation of atopy. It must be borne in mind that our sample was much smaller than that of Celiksoy et al.’s study (n=454). In contrast, our population of patients with X-linked Wiskott-Aldrich syndrome (WAS XL) was larger (n=252), although the prevalences of allergy and food allergy were only 4.4% and 2.8%, respectively. Our finding contradicts (i) statements about food allergy being a common feature of WAS (53, 54), and (ii) the results published by Lexmond et al. (36) in a notably smaller study of 15 patients, three (20%) of whom suffered from a clinically relevant food allergy.

There are various definitions of allergy in the literature, and many researchers refer to food allergy, allergic rhinitis, asthma, and atopic dermatitis. In the present review, the prevalence of any atopic condition would be 10.8% if one considered only eczematous dermatitis specified as atopic and 26.9% if one also considered unspecified “eczema”. In general, data on the frequency of atopic conditions in IEI are scarce, with estimates ranging from 10% to 28.8% (48). In an international study of atopic manifestations in IEI, El-Sayed et al. compared data from their own cohort with data from the USIDNET; the prevalence of atopy was 16.3% and 68.8%, respectively (55). The prevalence of food allergy in our review (5.6%) was higher than in El-Sayed et al.’s report (1.3%) and in the USIDNET data (0.1%), whereas the estimated prevalence in the general population ranges from 5% to 10% (18, 56). In contrast, the prevalence of allergic rhinitis in our review (1.8%) was lower than in El-Sayed et al.’s report (3%) and in the USIDNET data (8.2%) and well below the values of up to 25% reported for the general population (17); this might be due (at least partly) to under-reporting. The prevalence of asthma in our review (3.7%) was similar to that reported by El-Sayed et al. (3.6%) but the value in the USIDNET data was 46.9%. The prevalence of atopic dermatitis was 3.6% in El-Sayed et al.’s cohort and 23.7% in the USIDNET data. The prevalence of atopic dermatitis in our review depended on whether or not general mentions of “eczema” were considered, giving values of 24.6% and 4.2%, respectively. The interstudy differences in prevalence might be related to disparities in the methodology and in the proportions of IUIS groups.

In the general population, allergic rhinitis is more common than food allergy (17, 18). In our systematic review, however, the reported prevalence of allergic rhinitis was relatively low. This may reflect under-reporting in the literature and the predominance of pediatric cases, as severe IEI usually manifest early in life and are reported first. In accordance with the concept of the atopic march, children would be expected to develop food allergies first, while manifesting allergic rhinitis later. In the general population, atopic dermatitis is more prevalent than food allergy in children but less so in adults (15, 16). In our analysis, food allergy prevalence exceeded that of explicitly reported atopic dermatitis, which may be explained by inconsistent reporting of eczema subtypes in IEI, as authors often did not specify whether the eczema was “atopic”. Conversely, when combining atopic dermatitis with unspecified eczema, the prevalence (24.6%) clearly exceeded that of food allergy (5.6%), though this value may be exaggerated by inclusion of non-atopic eczema.

Beyond these methodological aspects, immunological factors may contribute to the predominance of food allergy in IEI. Defects in epithelial barrier function, impaired induction of oral tolerance, regulatory T cell deficiency, and mucosal microbiota dysbiosis are likely to favor allergic sensitization via the gastrointestinal tract (3, 48). Hyper-IgE states (e.g., in DOCK8 or CARD11 deficiency) are also recognized to accentuate food allergy as a clinical manifestation. Additionally, atypical mechanisms may mimic such states: in our own recent work, we described neutralizing anti-IL-6 autoantibodies in a patient with immune dysregulation polyendocrinopathy enteropathy X-linked (IPEX) syndrome (57), which likely induced a hyper-IgE-like phenotype with prominent food allergy, despite the absence of overt Th2 skewing. Taken together, these observations suggest that in IEI, food allergy may result not only from classical Th2 skewing but also from disease-specific immune dysregulation, including autoantibody-mediated modulation of the cytokine network.

Besides IgE-dependent allergy, several forms of IgE-independent allergy have been identified. An example of a food allergen-triggered disease is eosinophilic esophagitis, which appears to be a Th2-driven inflammation rather than an IgE-mediated disease and is frequently associated with other atopic conditions (58). On the other hand, the pathophysiology of food-protein induced enterocolitis syndrome (FPIES), a non-IgE, cell-mediated food allergy characterized by delayed onset vomiting and diarrhea and in severe cases, clinically relevant hypotension and marked dehydration (59–61), is suspected to involve antigen-specific T-cell responses and activation of innate immune cells (62). Furthermore, non-IgE-mediated hypersensitivity reactions to drugs, such as complement activation-related pseudo-allergy (CARPA), can present with symptoms resembling classical allergies, including anaphylactoid reactions (63, 64).

In the present study, we systematically reviewed the literature on the overlap between allergy and IEI. By using (i) a web application developed specifically for the creation of our database and (ii) HPO phenotype codes for data extraction, we recorded and extracted data in a standardized manner and produced an individual profile for each patient. This method will facilitate further expansion of the database and thus updating of the results. During the publication selection process, we sought to avoid the multiple inclusion of patients described in several different publications. By considering only the original publication, we eliminated a source of bias towards patients described in several reports.

Nonetheless, our review had some limitations. Firstly, we were unable to draw firm conclusions about the overall prevalence of allergy in IEI because cases of IEI presenting without immune dysregulation were not included. Secondly, the review’s design meant that publication bias is inherent: whether or not a case report is published depends on many factors, with rare and atypical case presentations being favored. Similarly, the presence of allergy may not have been reported or investigated at all if it was of no relevance to a study’s principal objective. As with genetic testing for IEI, allergy testing may prove more difficult in regions in which diagnostic resources are limited. Under-reporting of allergy can therefore be suspected, and our quantitative results should be interpreted with caution. In this regard, the apparent absence of allergic manifestations in LRBA deficiency – a disorder characterized by impaired regulatory T cell function and immune dysregulation – may at least in part reflect under-reporting. Furthermore, the spectrum and prevalence of observed allergies may depend on the age group and geographic region (24, 65), neither of which were considered in our analysis, as information on age at symptom onset, country of origin as well as sex was missing for many patients. Lastly, the severity and clinical presentation of allergies were not addressed in our review.

Our literature review highlighted IEI (spread over several IUIS tables and IEI subgroups) associated with a high prevalence of allergy and thus emphasized the utility of viewing atopy-associated IEI as a distinct group. The term “primary atopic disorder” suggested by Lyons and Milner (7) appears to be appropriate, with the possibility of further grouping IEI according to the underlying atopic mechanism. Furthermore, our results may serve as a template upon which future studies of the prevalence, distribution, and nature of atopic manifestations in monogenic disorders can be conducted. This approach might reveal new areas of interest and provide a better understanding of immune system function and dysfunction.

Our evidence-based overview of allergy in IEI highlighted disorders with particularly high or low prevalences of allergy. The results may raise clinicians’ awareness of these IEI, which might not be diagnosed and treated rapidly if allergic conditions constitute the predominant initial signs and symptoms (66). Furthermore, our findings should remind clinicians treating patients with IEI to determine whether clinical allergy is present; if other manifestations (such as infection or autoimmunity) are the primary focus, allergies might be left untreated. Our present work also shows why it is important to provide detailed clinical reports on patients with IEI. Atopic conditions may be the initial manifestation of an IEI. Conversely, when infectious or immune dysregulation symptoms are prominent, allergies need to be diagnosed and treated. Lastly, we hope that this review will foster future research on the mechanisms behind these disorders and, ultimately, on new approaches for treating allergic disease.

## Data Availability

The original contributions presented in the study are included in the article/**Supplementary Material**. Further inquiries can be directed to the corresponding author.
