# Supplementary material for: Allergic manifestations in inborn errors of immunity: a systematic scoping review
Source: Front Immunol. 2025 Oct 10;16:1666600. doi: 10.3389/fimmu.2025.1666600 (PMC12549635; doi:10.3389/fimmu.2025.1666600)
Supplement: Supplementary file 1 [file DataSheet1.docx]

Supplementary Material

# Supplementary Table S1

**Supplementary Table S1**. Inborn errors of immunity (IEI) subgroup abbreviations that appear in the manuscript and the Supplementary Figures, with their respective definitions. Usually, a single IEI subgroup corresponds to a single IUIS sub-table. When several IUIS sub-tables were similar in nature, they were pooled. In total, 37 IUIS sub-tables are represented by 27 subgroups.

| **Abbreviation  (as in figures)** | **Contained IUIS sub-tables (digit)** | **Contained IUIS sub-tables (name)** |
| --- | --- | --- |
| SCID T-B+ | 1.1 | T-B+ SCID |
| SCID T-B- | 1.2 | T-B- SCID |
| CID | 1.3 | CID, generally less profound than SCID |
| CID-Tc | 2.1 | Immunodeficiency with congenital thrombocytopenia |
| CID-DNA | 2.2 | DNA repair defects other than those listed in Table 1 |
| CID-Thymic | 2.3 | Thymic defects with additional congenital anomalies |
| CID-Oss | 2.4 | Immuno-osseous dysplasia |
| HIES | 2.5 | Hyper IgE syndromes (HIES) |
| CID-EDA | 2.7 | Anhidrotic ectodermodysplasia with immunodeficency (EDA-ID) |
| CID-Ca | 2.8 | Calcium channel defects |
| CID-other | 2.6 | Defects of vitamin B12 and folate metabolism |
|  | 2.9 | Other defects of CID with associated features |
| PAD-Agamma | 3.1 | Severe reduction in all serum immunoglobulin isotypes with profoundly decreased or absent B cells, agammaglobulinemia |
| PAD-CVID | 3.2 | Severe reduction in at least 2 serum immunoglobulin isotypes with normal or low number of B cells, CVID phenotype |
| PAD-other | 3.3 | Severe reduction in serum IgG and IgA with normal/elevated IgM and normal numbers of B cells, hyper IgM |
|  | 3.4 | Isotype, light chain, or functional deficiencies with generally normal numbers of B cells |
| PIRD-FHL | 4.1 | Familial hemophagocytic lymphohistiocytosis (FHL syndromes) |
|  | 4.2 | FHL syndromes with hypopigmentation |
| PIRD-Treg | 4.3 | Regulatory T cell defects |
| PIRD-AI | 4.4 | Autoimmunity with or without lymphoproliferation |
| PIRD-colitis | 4.5 | Immune dysregulation with colitis |
| PIRD-ALPS | 4.6 | Autoimmune lymphoproliferative syndrome (ALPS) |
| PIRD-EBV | 4.7 | Susceptibility to Epstein-Barr virus and lymphoproliferative conditions |
| Phagocyte | 5.1 | Congenital neutropenias |
|  | 5.2 | Defects of motility |
|  | 5.3 | Defects of respiratory burst |
|  | 5.4 | Other non-lymphoid defects |
| Innate | 6.1 | Mendelian susceptibility to mycobacterial disease |
|  | 6.3 | Predisposition to severe viral infection |
|  | 6.6 | Predisposition to mucocutaneous candidiasis |
|  | 6.7 | TLR signalling pathway deficiency with bacterial susceptibility |
|  | 6.8 | Other IEI related to non-hematopoietic tissues |
| AID-IFN1 | 7.1 | Type 1 interferonopathies |
| AID-Infl | 7.2 | Defects affecting the inflammasome |
| AID-Noninfl | 7.3 | Non-inflammasome related conditions |
| Complement | 8 | Complement deficiencies |
| BMF | 9 | Bone marrow failure |

# Supplementary Figures

**
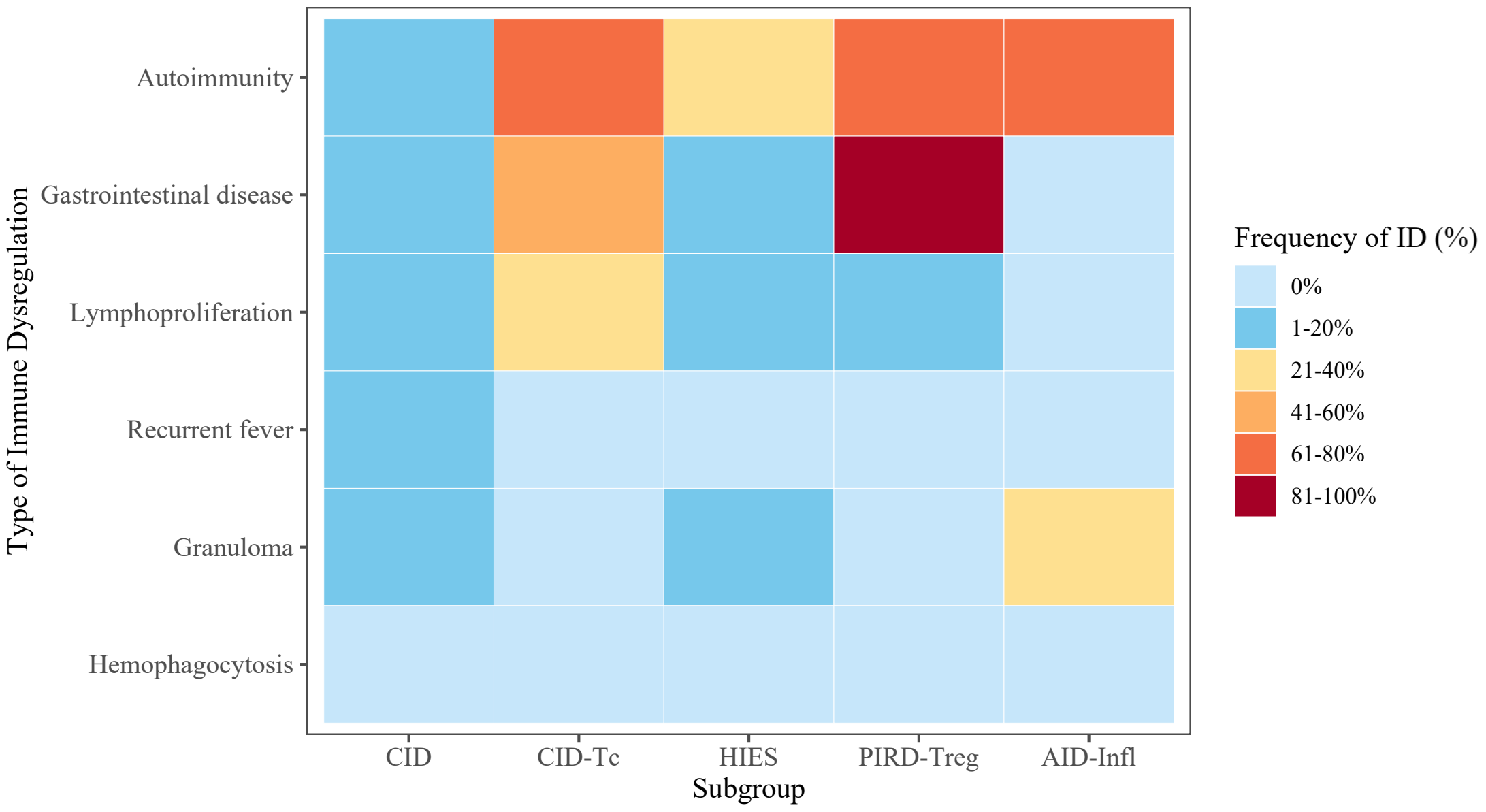
Supplementary Figure S1**. Frequency of immune dysregulation (ID) among patients with allergy, by subgroup. Only subgroups with at least 10 allergy patients are shown. The subgroups are defined in Supplementary Table S1.


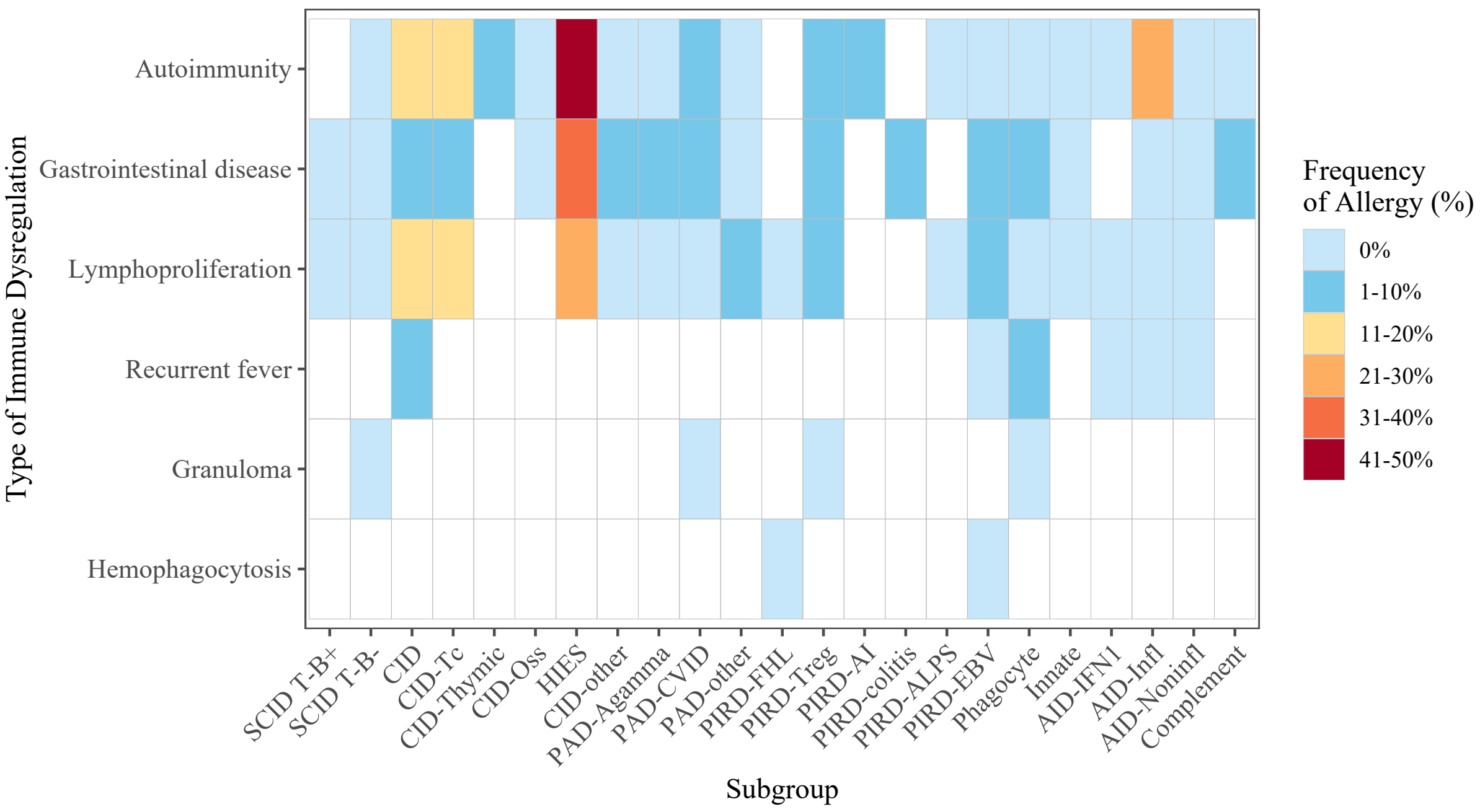
**Supplementary Figure S2**. Frequency of allergy among patients with immune dysregulation, by subgroup. Data are only shown if there were at least 10 patients with the immune dysregulation. Subgroups with less than 10 patients for each type of immune dysregulation are not displayed. The subgroups are defined in Supplementary Table S1.


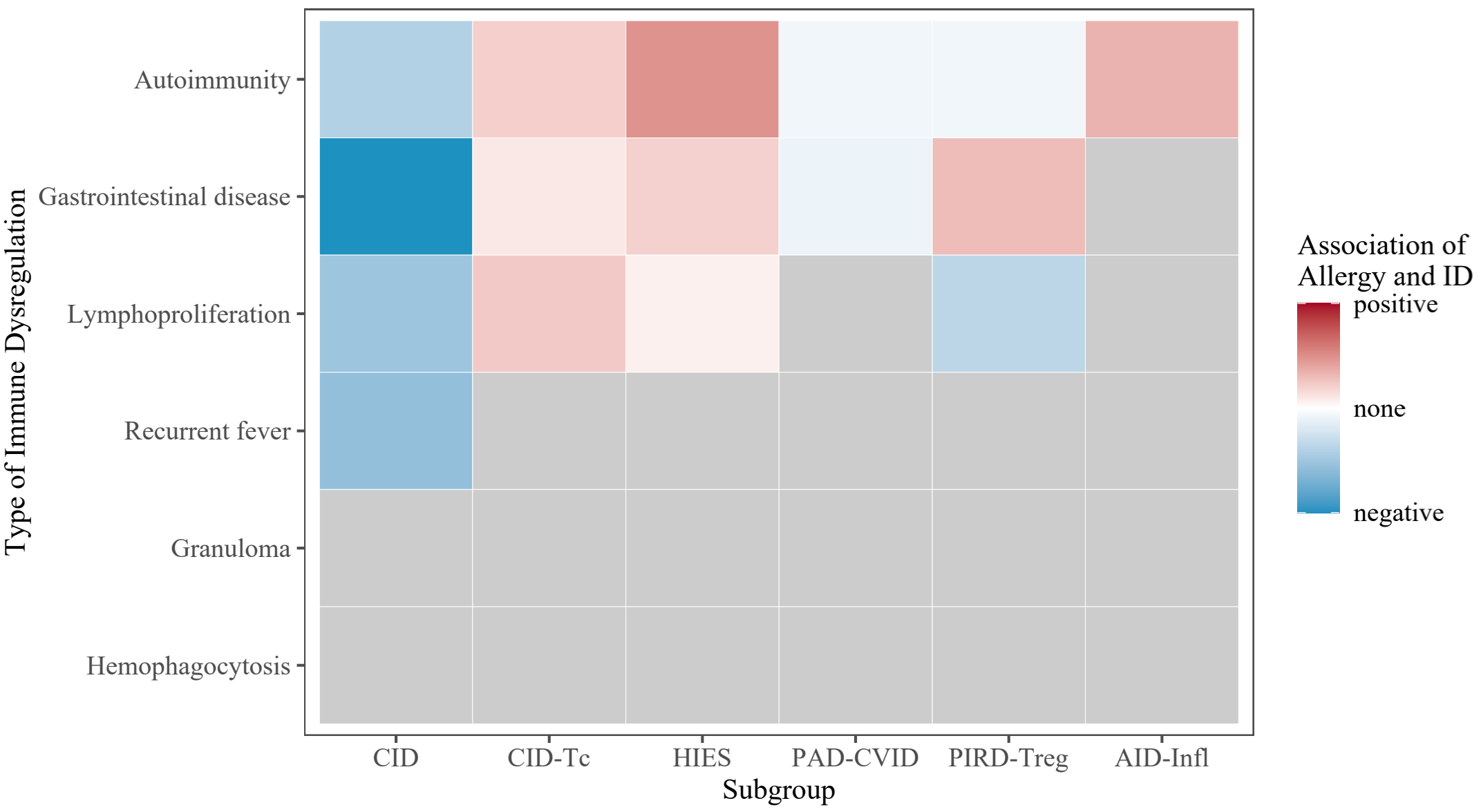


**Supplementary Figure S3**. The association between allergy and immune dysregulation (ID), by subgroup. Only subgroups with at least five allergy patients and five non-allergy patients were considered. The degree of association is shown only if there were at least 10 patients with the type of immune dysregulation and 10 patients without. The subgroups are defined in Supplementary Table S1.

# Search strategy

**Database: Embase**

Search from inception until February 4^th^, 2021. Applied limits: humans, article, article in press, conference paper, letter.

Total number of records found: 576

Search algorithm:

('primary immune regulatory disorders' OR 'inborn errors of immunity' OR 'primary immunodeficiency diseases' OR 'primary immunodeficiency' OR 'genetic immunodeficiency') AND (granuloma:ti,ab OR autoimmunity:ti,ab OR autoimmune:ti,ab OR autoinflammation:ti,ab OR 'recurring fever':ti,ab OR 'periodic fever':ti,ab OR 'chronic inflammation':ti,ab OR eczema:ti,ab OR rash:ti,ab OR lymphoproliferation:ti,ab OR lymphadenopathy:ti,ab OR splenomegaly:ti,ab OR hepatosplenomegaly:ti,ab OR 'inflammatory bowel disease':ti,ab OR enteropathy:ti,ab OR 'chronic diarrhea':ti,ab OR 'intestinal disease':ti,ab OR allergy:ti,ab) AND ('10p13 p14':ti,ab OR 14q32:ti,ab OR 22q11.2:ti,ab OR acd:ti,ab OR 'acd gene'/exp OR acp5:ti,ab OR 'acp5 gene'/exp OR actb:ti,ab OR 'actb gene'/exp OR ada:ti,ab OR 'ada gene'/exp OR ada2:ti,ab OR 'ada2 gene'/exp OR adam17:ti,ab OR 'adam17 gene'/exp OR adar:ti,ab OR 'adar gene'/exp OR adar1:ti,ab OR 'adar1 gene'/exp OR aicda:ti,ab OR 'aicda gene'/exp OR aire:ti,ab OR 'aire gene'/exp OR ak2:ti,ab OR 'ak2 gene'/exp OR alpi:ti,ab OR 'alpi gene'/exp OR ap1s3:ti,ab OR 'ap1s3 gene'/exp OR ap3b1:ti,ab OR 'ap3b1 gene'/exp OR ap3d1:ti,ab OR 'ap3d1 gene' OR apol1:ti,ab OR 'apol1 gene'/exp OR arhgef1:ti,ab OR 'arhgef1 gene' OR arpc1b:ti,ab OR 'arpc1b gene'/exp OR atm:ti,ab OR 'atm gene'/exp OR atp6ap1:ti,ab OR 'atp6ap1 gene'/exp OR b2m:ti,ab OR 'b2m gene'/exp OR bach2:ti,ab OR 'bach2 gene'/exp OR baffr:ti,ab OR 'baffr gene' OR bcl10:ti,ab OR 'bcl10 gene'/exp OR bcl11b:ti,ab OR 'bcl11b gene'/exp OR blm:ti,ab OR 'blm gene'/exp OR blnk:ti,ab OR 'blnk gene'/exp OR brca1:ti,ab OR 'brca1 gene'/exp OR brca2:ti,ab OR 'brca2 gene'/exp OR brip1:ti,ab OR 'brip1 gene'/exp OR btk:ti,ab OR 'btk gene'/exp OR c1q:ti,ab OR 'c1q gene'/exp OR c1qa:ti,ab OR 'c1qa gene'/exp OR c1qb:ti,ab OR 'c1qb gene'/exp OR c1qc:ti,ab OR 'c1qc gene'/exp OR c1r:ti,ab OR 'c1r gene'/exp OR c1s:ti,ab OR 'c1s gene'/exp OR c2:ti,ab OR 'c2 gene'/exp OR c2bp1:ti,ab OR 'c2bp1 gene' OR c3:ti,ab OR 'c3 gene'/exp OR c4:ti,ab OR 'c4 gene'/exp OR c4a:ti,ab OR 'c4a gene'/exp OR c4b:ti,ab OR 'c4b gene'/exp OR c5:ti,ab OR 'c5 gene'/exp OR c6:ti,ab OR 'c6 gene'/exp OR c7:ti,ab OR 'c7 gene'/exp OR c8a:ti,ab OR 'c8a gene' OR c8b:ti,ab OR 'c8b gene' OR c8g:ti,ab OR 'c8g gene' OR c9:ti,ab OR 'c9 gene'/exp OR card11:ti,ab OR 'card11 gene'/exp OR card14:ti,ab OR 'card14 gene'/exp OR card15:ti,ab OR 'card15 gene'/exp OR card9:ti,ab OR 'card9 gene'/exp OR carmil2:ti,ab OR 'carmil2 gene' OR casp10:ti,ab OR 'casp10 gene'/exp OR casp8:ti,ab OR 'casp8 gene'/exp OR ccbe1:ti,ab OR 'ccbe1 gene'/exp OR cd19:ti,ab OR 'cd19 gene'/exp OR cd20:ti,ab OR 'cd20 gene'/exp OR cd21:ti,ab OR 'cd21 gene' OR cd247:ti,ab OR 'cd247 gene'/exp OR cd27:ti,ab OR 'cd27 gene'/exp OR cd3d:ti,ab OR 'cd3d gene'/exp OR cd3e:ti,ab OR 'cd3e gene'/exp OR cd3g:ti,ab OR 'cd3g gene'/exp OR cd3z:ti,ab OR 'cd3z gene' OR cd40:ti,ab OR 'cd40 gene'/exp OR cd40lg:ti,ab OR 'cd40lg gene'/exp OR cd46:ti,ab OR 'cd46 gene'/exp OR cd55:ti,ab OR 'cd55 gene'/exp OR cd59:ti,ab OR 'cd59 gene'/exp OR cd70:ti,ab OR 'cd70 gene'/exp OR cd79a:ti,ab OR 'cd79a gene'/exp OR cd79b:ti,ab OR 'cd79b gene'/exp OR cd81:ti,ab OR 'cd81 gene'/exp OR cd8a:ti,ab OR 'cd8a gene'/exp OR cdca7:ti,ab OR 'cdca7 gene'/exp OR cebpe:ti,ab OR 'cebpe gene'/exp OR cecr1:ti,ab OR 'cecr1 gene'/exp OR cfb:ti,ab OR 'cfb gene'/exp OR cfd:ti,ab OR 'cfd gene'/exp OR cfh:ti,ab OR 'cfh gene'/exp OR cfhr:ti,ab OR 'cfhr gene' OR cfhr1:ti,ab OR 'cfhr1 gene'/exp OR cfhr2:ti,ab OR 'cfhr2 gene'/exp OR cfhr3:ti,ab OR 'cfhr3 gene'/exp OR cfhr4:ti,ab OR 'cfhr4 gene'/exp OR cfhr5:ti,ab OR 'cfhr5 gene'/exp OR cfi:ti,ab OR 'cfi gene'/exp OR cfp:ti,ab OR 'cfp gene'/exp OR cftr:ti,ab OR 'cftr gene'/exp OR chd7:ti,ab OR 'chd7 gene'/exp OR cias1:ti,ab OR 'cias1 gene'/exp OR cib1:ti,ab OR 'cib1 gene' OR ciita:ti,ab OR 'ciita gene'/exp OR clcn7:ti,ab OR 'clcn7 gene'/exp OR clpb:ti,ab OR 'clpb gene'/exp OR copa:ti,ab OR 'copa gene'/exp OR coro1a:ti,ab OR 'coro1a gene'/exp OR cr2:ti,ab OR 'cr2 gene'/exp OR csf2ra:ti,ab OR 'csf2ra gene'/exp OR csf2rb:ti,ab OR 'csf2rb gene'/exp OR csf3r:ti,ab OR 'csf3r gene'/exp OR ctc1:ti,ab OR 'ctc1 gene'/exp OR ctla4:ti,ab OR 'ctla4 gene'/exp OR ctps1:ti,ab OR 'ctps1 gene' OR ctsc:ti,ab OR 'ctsc gene'/exp OR cxcr4:ti,ab OR 'cxcr4 gene'/exp OR cyba:ti,ab OR 'cyba gene'/exp OR cybb:ti,ab OR 'cybb gene'/exp OR cybc1:ti,ab OR 'cybc1 gene' OR dbr1:ti,ab OR 'dbr1 gene' OR dclre1c:ti,ab OR 'dclre1c gene'/exp OR def6:ti,ab OR 'def6 gene' OR dkc1:ti,ab OR 'dkc1 gene'/exp OR dnajc21:ti,ab OR 'dnajc21 gene'/exp OR dnase1l3:ti,ab OR 'dnase1l3 gene'/exp OR dnase2:ti,ab OR 'dnase2 gene' OR dnmt3b:ti,ab OR 'dnmt3b gene'/exp OR dock2:ti,ab OR 'dock2 gene'/exp OR dock8:ti,ab OR 'dock8 gene'/exp OR efl1:ti,ab OR 'efl1 gene' OR elane:ti,ab OR 'elane gene'/exp OR epg5:ti,ab OR 'epg5 gene'/exp OR erbb2ip:ti,ab OR 'erbb2ip gene' OR erbin:ti,ab OR 'erbin gene' OR ercc4:ti,ab OR 'ercc4 gene'/exp OR ercc6l2:ti,ab OR 'ercc6l2 gene' OR extl3:ti,ab OR 'extl3 gene'/exp OR faap24:ti,ab OR 'faap24 gene' OR fadd:ti,ab OR 'fadd gene'/exp OR fanca:ti,ab OR 'fanca gene'/exp OR fancb:ti,ab OR 'fancb gene'/exp OR fancc:ti,ab OR 'fancc gene'/exp OR fancd2:ti,ab OR 'fancd2 gene'/exp OR fance:ti,ab OR 'fance gene'/exp OR fancf:ti,ab OR 'fancf gene'/exp OR fancg:ti,ab OR 'fancg gene'/exp OR fanci:ti,ab OR 'fanci gene'/exp OR fancl:ti,ab OR 'fancl gene'/exp OR fancm:ti,ab OR 'fancm gene'/exp OR fas:ti,ab OR 'fas gene'/exp OR faslg:ti,ab OR 'faslg gene'/exp OR fat4:ti,ab OR 'fat4 gene'/exp OR fcgr3a:ti,ab OR 'fcgr3a gene'/exp OR fcho1:ti,ab OR 'fcho1 gene' OR fcn3:ti,ab OR 'fcn3 gene'/exp OR fermt1:ti,ab OR 'fermt1 gene'/exp OR fermt3:ti,ab OR 'fermt3 gene'/exp OR foxn1:ti,ab OR 'foxn1 gene'/exp OR foxp3:ti,ab OR 'foxp3 gene'/exp OR fpr1:ti,ab OR 'fpr1 gene'/exp OR g6pc3:ti,ab OR 'g6pc3 gene'/exp OR g6pd:ti,ab OR 'g6pd gene'/exp OR g6pt1:ti,ab OR 'g6pt1 gene' OR gata2:ti,ab OR 'gata2 gene'/exp OR gcs1:ti,ab OR 'gcs1 gene' OR gfi1:ti,ab OR 'gfi1 gene'/exp OR gins1:ti,ab OR 'gins1 gene' OR havcr2:ti,ab OR 'havcr2 gene'/exp OR hax1:ti,ab OR 'hax1 gene'/exp OR hells:ti,ab OR 'hells gene'/exp OR hmox:ti,ab OR 'hmox gene' OR hmox1:ti,ab OR 'hmox1 gene'/exp OR hoil1:ti,ab OR 'hoil1 gene' OR hoip:ti,ab OR 'hoip gene' OR hyou1:ti,ab OR 'hyou1 gene'/exp OR icos:ti,ab OR 'icos gene'/exp OR icoslg:ti,ab OR 'icoslg gene'/exp OR ifih1:ti,ab OR 'ifih1 gene'/exp OR ifnar1:ti,ab OR 'ifnar1 gene'/exp OR ifnar2:ti,ab OR 'ifnar2 gene'/exp OR ifngr1:ti,ab OR 'ifngr1 gene'/exp OR ifngr2:ti,ab OR 'ifngr2 gene'/exp OR ighm:ti,ab OR 'ighm gene'/exp OR igkc:ti,ab OR 'igkc gene'/exp OR igll1:ti,ab OR 'igll1 gene'/exp OR ikba:ti,ab OR 'ikba gene'/exp OR ikbkb:ti,ab OR 'ikbkb gene'/exp OR ikbkg:ti,ab OR 'ikbkg gene'/exp OR ikzf1:ti,ab OR 'ikzf1 gene'/exp OR il10:ti,ab OR 'il10 gene'/exp OR il10ra:ti,ab OR 'il10ra gene'/exp OR il10rb:ti,ab OR 'il10rb gene'/exp OR il12b:ti,ab OR 'il12b gene'/exp OR il12rb1:ti,ab OR 'il12rb1 gene'/exp OR il12rb2:ti,ab OR 'il12rb2 gene'/exp OR il17f:ti,ab OR 'il17f gene'/exp OR il17ra:ti,ab OR 'il17ra gene'/exp OR il17rc:ti,ab OR 'il17rc gene'/exp OR il18bp:ti,ab OR 'il18bp gene' OR il1rn:ti,ab OR 'il1rn gene'/exp OR il21:ti,ab OR 'il21 gene'/exp OR il21r:ti,ab OR 'il21r gene'/exp OR il23r:ti,ab OR 'il23r gene'/exp OR il2ra:ti,ab OR 'il2ra gene'/exp OR il2rb:ti,ab OR 'il2rb gene'/exp OR il2rg:ti,ab OR 'il2rg gene'/exp OR il36rn:ti,ab OR 'il36rn gene'/exp OR il6r:ti,ab OR 'il6r gene'/exp OR il6st:ti,ab OR 'il6st gene'/exp OR il7r:ti,ab OR 'il7r gene'/exp OR ino80:ti,ab OR 'ino80 gene'/exp OR irak1:ti,ab OR 'irak1 gene'/exp OR irak4:ti,ab OR 'irak4 gene'/exp OR irf2bp2:ti,ab OR 'irf2bp2 gene'/exp OR irf3:ti,ab OR 'irf3 gene'/exp OR irf4:ti,ab OR 'irf4 gene'/exp OR irf7:ti,ab OR 'irf7 gene'/exp OR irf8:ti,ab OR 'irf8 gene'/exp OR irf9:ti,ab OR 'irf9 gene'/exp OR isg15:ti,ab OR 'isg15 gene'/exp OR itch:ti,ab OR 'itch gene'/exp OR itgb2:ti,ab OR 'itgb2 gene'/exp OR itk:ti,ab OR 'itk gene'/exp OR jagn1:ti,ab OR 'jagn1 gene' OR jak1:ti,ab OR 'jak1 gene'/exp OR jak3:ti,ab OR 'jak3 gene'/exp OR kdm6a:ti,ab OR 'kdm6a gene'/exp OR kmt2a:ti,ab OR 'kmt2a gene'/exp OR kmt2d:ti,ab OR 'kmt2d gene'/exp OR lamtor2:ti,ab OR 'lamtor2 gene' OR lat:ti,ab OR 'lat gene'/exp OR lck:ti,ab OR 'lck gene'/exp OR lig1:ti,ab OR 'lig1 gene'/exp OR lig4:ti,ab OR 'lig4 gene'/exp OR lpin2:ti,ab OR 'lpin2 gene'/exp OR lrba:ti,ab OR 'lrba gene'/exp OR lyst:ti,ab OR 'lyst gene'/exp OR mad2l2:ti,ab OR 'mad2l2 gene'/exp OR magt1:ti,ab OR 'magt1 gene'/exp OR malt1:ti,ab OR 'malt1 gene'/exp OR map3k14:ti,ab OR 'map3k14 gene'/exp OR masp2:ti,ab OR 'masp2 gene'/exp OR mcm4:ti,ab OR 'mcm4 gene'/exp OR mefv:ti,ab OR 'mefv gene'/exp OR mkl1:ti,ab OR 'mkl1 gene'/exp OR mll2:ti,ab OR 'mll2 gene'/exp OR mogs:ti,ab OR 'mogs gene' OR mrtfa:ti,ab OR 'mrtfa gene' OR ms4a1:ti,ab OR 'ms4a1 gene'/exp OR msh6:ti,ab OR 'msh6 gene'/exp OR msn:ti,ab OR 'msn gene'/exp OR mthfd1:ti,ab OR 'mthfd1 gene'/exp OR mvk:ti,ab OR 'mvk gene'/exp OR myd88:ti,ab OR 'myd88 gene'/exp OR mysm1:ti,ab OR 'mysm1 gene'/exp OR nalp3:ti,ab OR 'nalp3 gene'/exp OR nbas:ti,ab OR 'nbas gene'/exp OR nbn:ti,ab OR 'nbn gene'/exp OR nbs1:ti,ab OR 'nbs1 gene'/exp OR ncf1:ti,ab OR 'ncf1 gene'/exp OR ncf2:ti,ab OR 'ncf2 gene'/exp OR ncf4:ti,ab OR 'ncf4 gene'/exp OR ncstn:ti,ab OR 'ncstn gene'/exp OR nemo:ti,ab OR 'nemo gene'/exp OR nfat5:ti,ab OR 'nfat5 gene'/exp OR nfe2l2:ti,ab OR 'nfe2l2 gene'/exp OR nfkb1:ti,ab OR 'nfkb1 gene'/exp OR nfkb2:ti,ab OR 'nfkb2 gene'/exp OR nfkbia:ti,ab OR 'nfkbia gene'/exp OR nhej1:ti,ab OR 'nhej1 gene'/exp OR nhp2:ti,ab OR 'nhp2 gene'/exp OR nlrc4:ti,ab OR 'nlrc4 gene'/exp OR nlrp1:ti,ab OR 'nlrp1 gene'/exp OR nlrp12:ti,ab OR 'nlrp12 gene'/exp OR nlrp3:ti,ab OR 'nlrp3 gene'/exp OR nod2:ti,ab OR 'nod2 gene'/exp OR nola2:ti,ab OR 'nola2 gene' OR nola3:ti,ab OR 'nola3 gene' OR nop10:ti,ab OR 'nop10 gene'/exp OR nsmce3:ti,ab OR 'nsmce3 gene' OR oas1:ti,ab OR 'oas1 gene'/exp OR orai1:ti,ab OR 'orai1 gene'/exp OR ostm1:ti,ab OR 'ostm1 gene'/exp OR otulin:ti,ab OR 'otulin gene' OR palb2:ti,ab OR 'palb2 gene'/exp OR parn:ti,ab OR 'parn gene'/exp OR pepd:ti,ab OR 'pepd gene'/exp OR pgm3:ti,ab OR 'pgm3 gene'/exp OR pik3cd:ti,ab OR 'pik3cd gene'/exp OR pik3r1:ti,ab OR 'pik3r1 gene'/exp OR plcg2:ti,ab OR 'plcg2 gene'/exp OR plekhm1:ti,ab OR 'plekhm1 gene'/exp OR pms2:ti,ab OR 'pms2 gene'/exp OR pnp:ti,ab OR 'pnp gene'/exp OR pola1:ti,ab OR 'pola1 gene'/exp OR pold1:ti,ab OR 'pold1 gene'/exp OR pold2:ti,ab OR 'pold2 gene'/exp OR pole:ti,ab OR 'pole gene'/exp OR pole1:ti,ab OR 'pole1 gene' OR pole2:ti,ab OR 'pole2 gene'/exp OR polr3a:ti,ab OR 'polr3a gene'/exp OR polr3c:ti,ab OR 'polr3c gene' OR polr3f:ti,ab OR 'polr3f gene' OR prf1:ti,ab OR 'prf1 gene'/exp OR prkcd:ti,ab OR 'prkcd gene'/exp OR prkdc:ti,ab OR 'prkdc gene'/exp OR psen:ti,ab OR 'psen gene' OR psen1:ti,ab OR 'psen1 gene'/exp OR psenen:ti,ab OR 'psenen gene'/exp OR psmb8:ti,ab OR 'psmb8 gene'/exp OR psmg2:ti,ab OR 'psmg2 gene' OR pstpip1:ti,ab OR 'pstpip1 gene'/exp OR pten:ti,ab OR 'pten gene'/exp OR ptprc:ti,ab OR 'ptprc gene'/exp OR pypaf1:ti,ab OR 'pypaf1 gene' OR rab27a:ti,ab OR 'rab27a gene'/exp OR rac2:ti,ab OR 'rac2 gene'/exp OR rad51:ti,ab OR 'rad51 gene'/exp OR rad51c:ti,ab OR 'rad51c gene'/exp OR rag1:ti,ab OR 'rag1 gene'/exp OR rag2:ti,ab OR 'rag2 gene'/exp OR ranbp2:ti,ab OR 'ranbp2 gene'/exp OR rasgrp1:ti,ab OR 'rasgrp1 gene'/exp OR rbck1:ti,ab OR 'rbck1 gene'/exp OR recql3:ti,ab OR 'recql3 gene' OR rel:ti,ab OR 'rel gene'/exp OR rela:ti,ab OR 'rela gene'/exp OR relb:ti,ab OR 'relb gene'/exp OR rfwd3:ti,ab OR 'rfwd3 gene' OR rfx5:ti,ab OR 'rfx5 gene'/exp OR rfxank:ti,ab OR 'rfxank gene'/exp OR rfxap:ti,ab OR 'rfxap gene' OR rhoh:ti,ab OR 'rhoh gene'/exp OR ripk1:ti,ab OR 'ripk1 gene'/exp OR rltpr:ti,ab OR 'rltpr gene' OR rmrp:ti,ab OR 'rmrp gene'/exp OR rnaseh2a:ti,ab OR 'rnaseh2a gene'/exp OR rnaseh2b:ti,ab OR 'rnaseh2b gene'/exp OR rnaseh2c:ti,ab OR 'rnaseh2c gene'/exp OR rnf168:ti,ab OR 'rnf168 gene'/exp OR rnf31:ti,ab OR 'rnf31 gene' OR rnu4atac:ti,ab OR 'rnu4atac gene'/exp OR rorc:ti,ab OR 'rorc gene'/exp OR rpsa:ti,ab OR 'rpsa gene'/exp OR rtel1:ti,ab OR 'rtel1 gene'/exp OR samd9:ti,ab OR 'samd9 gene'/exp OR samd9l:ti,ab OR 'samd9l gene'/exp OR samhd1:ti,ab OR 'samhd1 gene'/exp OR sbds:ti,ab OR 'sbds gene'/exp OR sec61a1:ti,ab OR 'sec61a1 gene' OR sema3e:ti,ab OR 'sema3e gene'/exp OR serping1:ti,ab OR 'serping1 gene'/exp OR sh2d1a:ti,ab OR 'sh2d1a gene'/exp OR sh3bp2:ti,ab OR 'sh3bp2 gene'/exp OR sh3kbp1:ti,ab OR 'sh3kbp1 gene' OR skiv2l:ti,ab OR 'skiv2l gene'/exp OR slc29a3:ti,ab OR 'slc29a3 gene'/exp OR slc35c1:ti,ab OR 'slc35c1 gene'/exp OR slc37a4:ti,ab OR 'slc37a4 gene'/exp OR slc39a7:ti,ab OR 'slc39a7 gene' OR slc46a1:ti,ab OR 'slc46a1 gene'/exp OR slc7a7:ti,ab OR 'slc7a7 gene'/exp OR slx4:ti,ab OR 'slx4 gene'/exp OR smarcal1:ti,ab OR 'smarcal1 gene'/exp OR smarcd2:ti,ab OR 'smarcd2 gene' OR snx10:ti,ab OR 'snx10 gene'/exp OR sp110:ti,ab OR 'sp110 gene'/exp OR spink5:ti,ab OR 'spink5 gene'/exp OR sppl2a:ti,ab OR 'sppl2a gene' OR srp54:ti,ab OR 'srp54 gene'/exp OR srp72:ti,ab OR 'srp72 gene' OR stat1:ti,ab OR 'stat1 gene'/exp OR stat2:ti,ab OR 'stat2 gene'/exp OR stat3:ti,ab OR 'stat3 gene'/exp OR stat5b:ti,ab OR 'stat5b gene'/exp OR stim1:ti,ab OR 'stim1 gene'/exp OR sting1:ti,ab OR 'sting1 gene' OR stk4:ti,ab OR 'stk4 gene' OR stn1:ti,ab OR 'stn1 gene'/exp OR stx11:ti,ab OR 'stx11 gene'/exp OR stxbp2:ti,ab OR 'stxbp2 gene'/exp OR taci:ti,ab OR 'taci gene'/exp OR tap1:ti,ab OR 'tap1 gene'/exp OR tap2:ti,ab OR 'tap2 gene'/exp OR tapbp:ti,ab OR 'tapbp gene'/exp OR taz:ti,ab OR 'taz gene'/exp OR tbk1:ti,ab OR 'tbk1 gene'/exp OR tbx1:ti,ab OR 'tbx1 gene'/exp OR tcf3:ti,ab OR 'tcf3 gene'/exp OR tcirg1:ti,ab OR 'tcirg1 gene'/exp OR tcn2:ti,ab OR 'tcn2 gene'/exp OR terc:ti,ab OR 'terc gene'/exp OR tert:ti,ab OR 'tert gene'/exp OR tfrc:ti,ab OR 'tfrc gene'/exp OR tgfb1:ti,ab OR 'tgfb1 gene'/exp OR tgfbr1:ti,ab OR 'tgfbr1 gene'/exp OR tgfbr2:ti,ab OR 'tgfbr2 gene'/exp OR thbd:ti,ab OR 'thbd gene'/exp OR ticam1:ti,ab OR 'ticam1 gene' OR tinf2:ti,ab OR 'tinf2 gene'/exp OR tirap:ti,ab OR 'tirap gene'/exp OR tlr3:ti,ab OR 'tlr3 gene'/exp OR tmc6:ti,ab OR 'tmc6 gene'/exp OR tmc8:ti,ab OR 'tmc8 gene'/exp OR tmem173:ti,ab OR 'tmem173 gene'/exp OR tnfaip3:ti,ab OR 'tnfaip3 gene'/exp OR tnfrsf11a:ti,ab OR 'tnfrsf11a gene'/exp OR tnfrsf13b:ti,ab OR 'tnfrsf13b gene'/exp OR tnfrsf13c:ti,ab OR 'tnfrsf13c gene'/exp OR tnfrsf1a:ti,ab OR 'tnfrsf1a gene'/exp OR tnfrsf4:ti,ab OR 'tnfrsf4 gene'/exp OR tnfrsf5:ti,ab OR 'tnfrsf5 gene' OR tnfrsf6:ti,ab OR 'tnfrsf6 gene' OR tnfrsf9:ti,ab OR 'tnfrsf9 gene'/exp OR tnfsf11:ti,ab OR 'tnfsf11 gene'/exp OR tnfsf12:ti,ab OR 'tnfsf12 gene'/exp OR tnfsf5:ti,ab OR 'tnfsf5 gene' OR tnfsf6:ti,ab OR 'tnfsf6 gene' OR tnfsf7:ti,ab OR 'tnfsf7 gene' OR top2b:ti,ab OR 'top2b gene'/exp OR tp53:ti,ab OR 'tp53 gene'/exp OR tpp1:ti,ab OR 'tpp1 gene'/exp OR tpp2:ti,ab OR 'tpp2 gene' OR trac:ti,ab OR 'trac gene'/exp OR traf3:ti,ab OR 'traf3 gene'/exp OR traf3ip2:ti,ab OR 'traf3ip2 gene'/exp OR trex1:ti,ab OR 'trex1 gene'/exp OR trim22:ti,ab OR 'trim22 gene'/exp OR trnt1:ti,ab OR 'trnt1 gene'/exp OR ttc37:ti,ab OR 'ttc37 gene'/exp OR ttc7a:ti,ab OR 'ttc7a gene'/exp OR tyk2:ti,ab OR 'tyk2 gene'/exp OR ube2t:ti,ab OR 'ube2t gene'/exp OR unc13d:ti,ab OR 'unc13d gene'/exp OR unc93b1:ti,ab OR 'unc93b1 gene'/exp OR ung:ti,ab OR 'ung gene'/exp OR usb1:ti,ab OR 'usb1 gene' OR usp18:ti,ab OR 'usp18 gene'/exp OR vps13b:ti,ab OR 'vps13b gene'/exp OR vps45:ti,ab OR 'vps45 gene' OR 'was gene'/exp OR wasp:ti,ab OR 'wasp gene'/exp OR wdr1:ti,ab OR 'wdr1 gene'/exp OR wipf1:ti,ab OR 'wipf1 gene'/exp OR 'wiskott aldrich syndrome':ti,ab OR wrap53:ti,ab OR 'wrap53 gene'/exp OR xiap:ti,ab OR 'xiap gene'/exp OR xrcc2:ti,ab OR 'xrcc2 gene'/exp OR xrcc9:ti,ab OR 'xrcc9 gene' OR zap70:ti,ab OR 'zap70 gene'/exp OR zbtb24:ti,ab OR 'zbtb24 gene'/exp OR znf341:ti,ab OR 'znf341 gene' OR pid:ti,ab OR 'pid gene' OR 'baff r':ti,ab OR 'baff-r gene' OR c4a+c4b:ti,ab OR 'c4a+c4b gene') NOT hiv

**Database: Scopus**

Search from inception until February 4^th^, 2021. Applied limits: article, conference paper, letter.

Total number of records found: 1361

Search Algorithm:

("primary immune regulatory disorders" OR "inborn errors of immunity" OR "Primary Immunodeficiency Diseases" OR "primary Immunodeficiency" OR "genetic Immunodeficiency") AND (TITLE-ABS(granuloma) OR TITLE- ABS(autoimmunity) OR TITLE-ABS( autoimmune) OR TITLE-ABS(autoinflammation) OR TITLE-ABS("recurring fever") OR TITLE-ABS("periodic fever") OR TITLE-ABS("chronic inflammation") OR TITLE-ABS(eczema) OR TITLE-ABS(rash) OR TITLE-ABS(lymphoproliferation) OR TITLE-ABS(lymphadenopathy) OR TITLE- ABS(splenomegaly) OR TITLE-ABS(hepatosplenomegaly) OR TITLE-ABS(" inflammatory bowel disease") OR TITLE-ABS(enteropathy) OR TITLE-ABS("chronic diarrhea") OR TITLE-ABS("intestinal disease") OR TITLE- ABS(allergy)) AND (TITLE-ABS(10p13-p14) OR TITLE-ABS(14q32) OR TITLE-ABS(22q11.2) OR TITLE-ABS( acd) OR TITLE-ABS(acp5) OR TITLE-ABS(actb) OR TITLE-ABS(ada) OR TITLE-ABS(ada2) OR TITLE- ABS(adam17) OR TITLE-ABS(adar) OR TITLE-ABS(adar1) OR TITLE-ABS(aicda) OR TITLE-ABS(aire) OR TITLE-ABS(ak2) OR TITLE-ABS(alpi) OR TITLE-ABS(ap1s3) OR TITLE-ABS(ap3b1) OR TITLE-ABS(ap3d1) OR TITLE-ABS(apol1) OR TITLE-ABS(arhgef1) OR TITLE-ABS(arpc1b) OR TITLE-ABS(atm) OR TITLE- ABS(atp6ap1) OR TITLE-ABS(b2m) OR TITLE-ABS(bach2) OR TITLE-ABS( baffr) OR TITLE-ABS("baff-r") OR TITLE-ABS(bcl10) OR TITLE-ABS(bcl11b) OR TITLE-ABS(blm) OR TITLE-ABS(blnk) OR TITLE-ABS(brca1) OR TITLE-ABS(brca2) OR TITLE-ABS(brip1) OR TITLE-ABS(btk) OR TITLE-ABS(c1q) OR TITLE-ABS(c1qa) OR TITLE-ABS(c1qb) OR TITLE-ABS(c1qc) OR TITLE-ABS(c1r) OR TITLE-ABS(c1s) OR TITLE-ABS(c2) OR TITLE-ABS( c2bp1) OR TITLE-ABS(c3) OR TITLE-ABS(c4) OR TITLE-ABS(c4a) OR TITLE-ABS("c4a+c4b") OR TITLE- ABS(c4b) OR TITLE-ABS(c5) OR TITLE-ABS(c6) OR TITLE-ABS(c7) OR TITLE-ABS(c8a) OR TITLE-ABS(c8b) OR TITLE-ABS(c8g) OR TITLE-ABS(c9) OR TITLE-ABS(card11) OR TITLE-ABS(card14) OR TITLE- ABS(card15) OR TITLE-ABS(card9) OR TITLE-ABS( carmil2) OR TITLE-ABS(casp10) OR TITLE-ABS(casp8) OR TITLE-ABS(ccbe1) OR TITLE-ABS(cd19) OR TITLE-ABS( cd20) OR TITLE-ABS(cd21) OR TITLE-ABS(cd247) OR TITLE-ABS(cd27) OR TITLE-ABS(cd3d) OR TITLE-ABS(cd3e) OR TITLE-ABS(cd3g) OR TITLE-ABS(cd3z) OR TITLE-ABS(cd40) OR TITLE-ABS(cd40lg) OR TITLE-ABS(cd46) OR TITLE-ABS( cd55) OR TITLE-ABS(cd59) OR TITLE-ABS(cd70) OR TITLE-ABS(cd79a) OR TITLE-ABS(cd79b) OR TITLE-ABS(cd81) OR TITLE- ABS(cd8a) OR TITLE-ABS(cdca7) OR TITLE-ABS(cebpe) OR TITLE-ABS(cecr1) OR TITLE-ABS(cfb) OR TITLE-ABS( cfd) OR TITLE-ABS(cfh) OR TITLE-ABS(cfhr) OR TITLE-ABS(cfhr1) OR TITLE-ABS(cfhr2) OR TITLE- ABS(cfhr3) OR TITLE-ABS(cfhr4) OR TITLE-ABS(cfhr5) OR TITLE-ABS(cfi) OR TITLE-ABS(cfp) OR TITLE- ABS(cftr) OR TITLE-ABS(chd7) OR TITLE-ABS(cias1) OR TITLE-ABS(cib1) OR TITLE-ABS(ciita) OR TITLE- ABS(clcn7) OR TITLE-ABS(clpb) OR TITLE-ABS(copa) OR TITLE-ABS(coro1a) OR TITLE-ABS(cr2) OR TITLE- ABS(csf2ra) OR TITLE-ABS(csf2rb) OR TITLE-ABS(csf3r) OR TITLE-ABS( ctc1) OR TITLE-ABS(ctla4) OR TITLE-ABS(ctps1) OR TITLE-ABS(ctsc) OR TITLE-ABS(cxcr4) OR TITLE-ABS(cyba) OR TITLE-ABS(cybb) OR TITLE-ABS(cybc1) OR TITLE-ABS(dbr1) OR TITLE-ABS(dclre1c) OR TITLE-ABS(def6) OR TITLE-ABS( dkc1) OR TITLE-ABS(dnajc21) OR TITLE-ABS(dnase1l3) OR TITLE-ABS(dnase2) OR TITLE-ABS(dnmt3b) OR TITLE-ABS( dock2) OR TITLE-ABS(dock8) OR TITLE-ABS(efl1) OR TITLE-ABS(elane) OR TITLE-ABS(epg5) OR TITLE-ABS(erbb2ip) OR TITLE-ABS(erbin) OR TITLE-ABS(ercc4) OR TITLE-ABS(ercc6l2) OR TITLE-ABS(extl3) OR TITLE-ABS(faap24) OR TITLE-ABS( fadd) OR TITLE-ABS(fanca) OR TITLE-ABS(fancb) OR TITLE- ABS(fancc) OR TITLE-ABS(fancd2) OR TITLE-ABS(fance) OR TITLE-ABS(fancf) OR TITLE-ABS(fancg) OR TITLE-ABS(fanci) OR TITLE-ABS(fancl) OR TITLE-ABS(fancm) OR TITLE-ABS( fas) OR TITLE-ABS(faslg) OR TITLE-ABS(fat4) OR TITLE-ABS(fcgr3a) OR TITLE-ABS(fcho1) OR TITLE-ABS(fcn3) OR TITLE-ABS(fermt1) OR TITLE-ABS(fermt3) OR TITLE-ABS(foxn1) OR TITLE-ABS(foxp3) OR TITLE-ABS(fpr1) OR TITLE-ABS( g6pc3) OR TITLE-ABS(g6pd) OR TITLE-ABS(g6pt1) OR TITLE-ABS(gata2) OR TITLE-ABS(gcs1) OR TITLE-ABS(gfi1) OR TITLE-ABS(gins1) OR TITLE-ABS(havcr2) OR TITLE-ABS(hax1) OR TITLE-ABS(hells) OR TITLE- ABS(hmox) OR TITLE-ABS( hmox1) OR TITLE-ABS(hoil1) OR TITLE-ABS(hoip) OR TITLE-ABS(hyou1) OR TITLE-ABS(icos) OR TITLE-ABS(icoslg) OR TITLE-ABS(ifih1) OR TITLE-ABS(ifnar1) OR TITLE-ABS(ifnar2) OR TITLE-ABS(ifngr1) OR TITLE-ABS(ifngr2) OR TITLE-ABS( ighm) OR TITLE-ABS(igkc) OR TITLE-ABS(igll1) OR TITLE-ABS(ikba) OR TITLE-ABS(ikbkb) OR TITLE-ABS(ikbkg) OR TITLE-ABS(ikzf1) OR TITLE-ABS(il10) OR TITLE-ABS(il10ra) OR TITLE-ABS(il10rb) OR TITLE-ABS(il12b) OR TITLE-ABS( il12rb1) OR TITLE-ABS(il12rb2) OR TITLE-ABS(il17f) OR TITLE-ABS(il17ra) OR TITLE-ABS(il17rc) OR TITLE-ABS(il18bp) OR TITLE-ABS(il1rn) OR TITLE-ABS(il21) OR TITLE-ABS(il21r) OR TITLE-ABS(il23r) OR TITLE-ABS(il2ra) OR TITLE-ABS(il2rb) OR TITLE-ABS(il2rg) OR TITLE-ABS(il36rn) OR TITLE-ABS(il6r) OR TITLE-ABS(il6st) OR TITLE-ABS(il7r) OR TITLE-ABS( ino80) OR TITLE-ABS(irak1) OR TITLE-ABS(irak4) OR TITLE-ABS(irf2bp2) OR TITLE-ABS(irf3) OR TITLE-ABS(irf4) OR TITLE-ABS(irf7) OR TITLE-ABS(irf8) OR TITLE-ABS(irf9) OR TITLE-ABS(isg15) OR TITLE- ABS(itch) OR TITLE-ABS(itgb2) OR TITLE-ABS(itk) OR TITLE-ABS(jagn1) OR TITLE-ABS(jak1) OR TITLE- ABS(jak3) OR TITLE-ABS(kdm6a) OR TITLE-ABS( kmt2a) OR TITLE-ABS(kmt2d) OR TITLE-ABS(lamtor2) OR TITLE-ABS(lat) OR TITLE-ABS(lck) OR TITLE-ABS(lig1) OR TITLE-ABS(lig4) OR TITLE-ABS(lpin2) OR TITLE- ABS(lrba) OR TITLE-ABS(lyst) OR TITLE-ABS(mad2l2) OR TITLE-ABS( magt1) OR TITLE-ABS(malt1) OR TITLE-ABS(map3k14) OR TITLE-ABS(masp2) OR TITLE-ABS(mcm4) OR TITLE-ABS( mefv) OR TITLE- ABS(mkl1) OR TITLE-ABS(mll2) OR TITLE-ABS(mogs) OR TITLE-ABS(mrtfa) OR TITLE-ABS(ms4a1) OR TITLE-ABS(msh6) OR TITLE-ABS(msn) OR TITLE-ABS(mthfd1) OR TITLE-ABS(mvk) OR TITLE-ABS(myd88) OR TITLE-ABS( mysm1) OR TITLE-ABS(nalp3) OR TITLE-ABS(nbas) OR TITLE-ABS(nbn) OR TITLE-ABS(nbs1) OR TITLE-ABS(ncf1) OR TITLE-ABS(ncf2) OR TITLE-ABS(ncf4) OR TITLE-ABS(ncstn) OR TITLE-ABS(nemo) OR TITLE-ABS(nfat5) OR TITLE-ABS( nfe2l2) OR TITLE-ABS(nfkb1) OR TITLE-ABS(nfkb2) OR TITLE- ABS(nfkbia) OR TITLE-ABS(nhej1) OR TITLE-ABS(nhp2) OR TITLE-ABS(nlrc4) OR TITLE-ABS(nlrp1) OR TITLE-ABS(nlrp12) OR TITLE-ABS(nlrp3) OR TITLE-ABS(nod2) OR TITLE-ABS( nola2) OR TITLE-ABS(nola3) OR TITLE-ABS(nop10) OR TITLE-ABS(nsmce3) OR TITLE-ABS(oas1) OR TITLE-ABS(orai1) OR TITLE- ABS(ostm1) OR TITLE-ABS(otulin) OR TITLE-ABS(palb2) OR TITLE-ABS(parn) OR TITLE-ABS(pepd) OR TITLE-ABS( pgm3) OR TITLE-ABS(pik3cd) OR TITLE-ABS(pik3r1) OR TITLE-ABS(plcg2) OR TITLE- ABS(plekhm1) OR TITLE-ABS( pms2) OR TITLE-ABS(pnp) OR TITLE-ABS(pola1) OR TITLE-ABS(pold1) OR TITLE-ABS(pold2) OR TITLE-ABS(pole) OR TITLE-ABS(pole1) OR TITLE-ABS(pole2) OR TITLE-ABS(polr3a) OR TITLE-ABS(polr3c) OR TITLE-ABS(polr3f) OR TITLE-ABS( prf1) OR TITLE-ABS(prkcd) OR TITLE- ABS(prkdc) OR TITLE-ABS(psen) OR TITLE-ABS(psen1) OR TITLE-ABS(psenen) OR TITLE-ABS(psmb8) OR TITLE-ABS(psmg2) OR TITLE-ABS(pstpip1) OR TITLE-ABS(pten) OR TITLE-ABS(ptprc) OR TITLE-ABS( pypaf1) OR TITLE-ABS(rab27a) OR TITLE-ABS(rac2) OR TITLE-ABS(rad51) OR TITLE-ABS(rad51c) OR TITLE-ABS( rag1) OR TITLE-ABS(rag2) OR TITLE-ABS(ranbp2) OR TITLE-ABS(rasgrp1) OR TITLE-ABS(rbck1) OR TITLE-ABS(recql3) OR TITLE-ABS(rel) OR TITLE-ABS(rela) OR TITLE-ABS(relb) OR TITLE-ABS(rfwd3) OR TITLE-ABS(rfx5) OR TITLE-ABS( rfxank) OR TITLE-ABS(rfxap) OR TITLE-ABS(rhoh) OR TITLE-ABS(ripk1) OR TITLE-ABS(rltpr) OR TITLE-ABS(rmrp) OR TITLE-ABS(rnaseh2a) OR TITLE-ABS(rnaseh2b) OR TITLE- ABS(rnaseh2c) OR TITLE-ABS(rnf168) OR TITLE-ABS(rnf31) OR TITLE-ABS(rnu4atac) OR TITLE-ABS(rorc) OR TITLE-ABS(rpsa) OR TITLE-ABS(rtel1) OR TITLE-ABS(samd9) OR TITLE-ABS( samd9l) OR TITLE- ABS(samhd1) OR TITLE-ABS(sbds) OR TITLE-ABS(sec61a1) OR TITLE-ABS(sema3e) OR TITLE-ABS( serping1) OR TITLE-ABS(sh2d1a) OR TITLE-ABS(sh3bp2) OR TITLE-ABS(sh3kbp1) OR TITLE- ABS(skiv2l) OR TITLE-ABS( slc29a3) OR TITLE-ABS(slc35c1) OR TITLE-ABS(slc37a4) OR TITLE-ABS(slc39a7) OR TITLE-ABS(slc46a1) OR TITLE-ABS( slc7a7) OR TITLE-ABS(slx4) OR TITLE-ABS(smarcal1) OR TITLE- ABS(smarcd2) OR TITLE-ABS(snx10) OR TITLE-ABS( sp110) OR TITLE-ABS(spink5) OR TITLE-ABS(sppl2a) OR TITLE-ABS(srp54) OR TITLE-ABS(srp72) OR TITLE-ABS(stat1) OR TITLE-ABS(stat2) OR TITLE-ABS(stat3) OR TITLE-ABS(stat5b) OR TITLE-ABS(stim1) OR TITLE-ABS(sting1) OR TITLE-ABS( stk4) OR TITLE-ABS(stn1) OR TITLE-ABS(stx11) OR TITLE-ABS(stxbp2) OR TITLE-ABS(taci) OR TITLE-ABS(tap1) OR TITLE-ABS(tap2) OR TITLE-ABS(tapbp) OR TITLE-ABS(taz) OR TITLE-ABS(tbk1) OR TITLE-ABS(tbx1) OR TITLE-ABS(tcf3) OR TITLE-ABS(tcirg1) OR TITLE-ABS(tcn2) OR TITLE-ABS(terc) OR TITLE-ABS(tert) OR TITLE-ABS(tfrc) OR TITLE-ABS( tgfb1) OR TITLE-ABS(tgfbr1) OR TITLE-ABS(tgfbr2) OR TITLE-ABS(thbd) OR TITLE-ABS(ticam1) OR TITLE-ABS(tinf2) OR TITLE-ABS(tirap) OR TITLE-ABS(tlr3) OR TITLE-ABS(tmc6) OR TITLE-ABS(tmc8) OR TITLE-ABS(tmem173) OR TITLE-ABS( tnfaip3) OR TITLE-ABS(tnfrsf11a) OR TITLE-ABS(tnfrsf13b) OR TITLE- ABS(tnfrsf13c) OR TITLE-ABS(tnfrsf1a) OR TITLE-ABS( tnfrsf4) OR TITLE-ABS(tnfrsf5) OR TITLE-ABS(tnfrsf6) OR TITLE-ABS(tnfrsf9) OR TITLE-ABS(tnfsf11) OR TITLE-ABS( tnfsf12) OR TITLE-ABS(tnfsf5) OR TITLE- ABS(tnfsf6) OR TITLE-ABS(tnfsf7) OR TITLE-ABS(top2b) OR TITLE-ABS(tp53) OR TITLE-ABS(tpp1) OR TITLE- ABS(tpp2) OR TITLE-ABS(trac) OR TITLE-ABS(traf3) OR TITLE-ABS(traf3ip2) OR TITLE-ABS( trex1) OR TITLE- ABS(trim22) OR TITLE-ABS(trnt1) OR TITLE-ABS(ttc37) OR TITLE-ABS(ttc7a) OR TITLE-ABS(tyk2) OR TITLE- ABS(ube2t) OR TITLE-ABS(unc13d) OR TITLE-ABS(unc93b1) OR TITLE-ABS(ung) OR TITLE-ABS(usb1) OR TITLE-ABS( usp18) OR TITLE-ABS(vps13b) OR TITLE-ABS(vps45) OR TITLE-ABS(wasp) OR TITLE-ABS(wdr1) OR TITLE-ABS(wipf1) OR TITLE-ABS("wiskott aldrich syndrome") OR TITLE-ABS(wrap53) OR TITLE-ABS(xiap) OR TITLE-ABS(xrcc2) OR TITLE-ABS( xrcc9) OR TITLE-ABS(zap70) OR TITLE-ABS(zbtb24) OR TITLE- ABS(znf341) OR TITLE-ABS(pid )) AND NOT HIV

**Database: Ovid**

Search from inception until February 4^th^, 2021. Applied limits: case report.

Total number of records found: 500

Search Algorithm:

('primary immune regulatory disorders' OR 'inborn errors of immunity' OR 'Primary Immunodeficiency Diseases' OR 'Primary immunodeficiency' OR 'Genetic immunodeficiency') NOT HIV AND (( granuloma ).ti,ab. or ( autoimmunity ).ti,ab. or ( autoimmune ).ti,ab. or ( autoinflammation ).ti,ab. or ( recurring fever ).ti,ab. or ( periodic fever ).ti,ab. or ( chronic inflammation ).ti,ab. or ( eczema ).ti,ab. or ( rash ).ti,ab. or ( lymphoproliferation ).ti,ab. or ( lymphadenopathy ).ti,ab. or ( splenomegaly ).ti,ab. or ( hepatosplenomegaly ).ti,ab. or ( inflammatory bowel disease ).ti,ab. or ( enteropathy ).ti,ab. or ( chronic diarrhea ).ti,ab. or ( intestinal disease ).ti,ab. or ( allergy ).ti,ab.) AND (( 10p13-p14 ).ti,ab. or ( 14q32 ).ti,ab. or ( 22q112 ).ti,ab. or ( acd ).ti,ab. or ( acp5 ).ti,ab. or ( actb ).ti,ab. or ( ada ).ti,ab. or ( ada2 ).ti,ab. or ( adam17 ).ti,ab. or ( adar ).ti,ab. or ( adar1 ).ti,ab. or ( aicda ).ti,ab. or ( aire ).ti,ab. or ( ak2 ).ti,ab. or ( alpi ).ti,ab. or ( ap1s3 ).ti,ab. or ( ap3b1 ).ti,ab. or ( ap3d1 ).ti,ab. or ( apol1 ).ti,ab. or ( arhgef1 ).ti,ab. or ( arpc1b ).ti,ab. or ( atm ).ti,ab. or ( atp6ap1 ).ti,ab. or ( b2m ).ti,ab. or ( bach2 ).ti,ab. or ( baffr ).ti,ab. or ( 'baff-r' ).ti,ab. or ( bcl10 ).ti,ab. or ( bcl11b ).ti,ab. or ( blm ).ti,ab. or ( blnk ).ti,ab. or ( brca1 ).ti,ab. or ( brca2 ).ti,ab. or ( brip1 ).ti,ab. or ( btk ).ti,ab. or ( c1q ).ti,ab. or ( c1qa ).ti,ab. or ( c1qb ).ti,ab. or ( c1qc ).ti,ab. or ( c1r ).ti,ab. or ( c1s ).ti,ab. or ( c2 ).ti,ab. or ( c2bp1 ).ti,ab. or ( c3 ).ti,ab. or ( c4 ).ti,ab. or ( c4a ).ti,ab. or ( 'c4a+c4b' ).ti,ab. or ( c4b ).ti,ab. or ( c5 ).ti,ab. or ( c6 ).ti,ab. or ( c7 ).ti,ab. or ( c8a ).ti,ab. or ( c8b ).ti,ab. or ( c8g ).ti,ab. or ( c9 ).ti,ab. or ( card11 ).ti,ab. or ( card14 ).ti,ab. or ( card15 ).ti,ab. or ( card9 ).ti,ab. or ( carmil2 ).ti,ab. or ( casp10 ).ti,ab. or ( casp8 ).ti,ab. or ( ccbe1 ).ti,ab. or ( cd19 ).ti,ab. or ( cd20 ).ti,ab. or ( cd21 ).ti,ab. or ( cd247 ).ti,ab. or ( cd27 ).ti,ab. or ( cd3d ).ti,ab. or ( cd3e ).ti,ab. or ( cd3g ).ti,ab. or ( cd3z ).ti,ab. or ( cd40 ).ti,ab. or ( cd40lg ).ti,ab. or ( cd46 ).ti,ab. or ( cd55 ).ti,ab. or ( cd59 ).ti,ab. or ( cd70 ).ti,ab. or ( cd79a ).ti,ab. or ( cd79b ).ti,ab. or ( cd81 ).ti,ab. or ( cd8a ).ti,ab. or ( cdca7 ).ti,ab. or ( cebpe ).ti,ab. or ( cecr1 ).ti,ab. or ( cfb ).ti,ab. or ( cfd ).ti,ab. or ( cfh ).ti,ab. or ( cfhr ).ti,ab. or ( cfhr1 ).ti,ab. or ( cfhr2 ).ti,ab. or ( cfhr3 ).ti,ab. or ( cfhr4 ).ti,ab. or ( cfhr5 ).ti,ab. or ( cfi ).ti,ab. or ( cfp ).ti,ab. or ( cftr ).ti,ab. or ( chd7 ).ti,ab. or ( cias1 ).ti,ab. or ( cib1 ).ti,ab. or ( ciita ).ti,ab. or ( clcn7 ).ti,ab. or ( clpb ) .ti,ab. or ( copa ).ti,ab. or ( coro1a ).ti,ab. or ( cr2 ).ti,ab. or ( csf2ra ).ti,ab. or ( csf2rb ).ti,ab. or ( csf3r ).ti,ab. or ( ctc1 ).ti,ab. or ( ctla4 ).ti,ab. or ( ctps1 ).ti,ab. or ( ctsc ).ti,ab. or ( cxcr4 ).ti,ab. or ( cyba ).ti,ab. or ( cybb ).ti,ab. or ( cybc1 ).ti,ab. or ( dbr1 ).ti,ab. or ( dclre1c ).ti,ab. or ( def6 ).ti,ab. or ( dkc1 ).ti,ab. or ( dnajc21 ).ti,ab. or ( dnase1l3 ).ti,ab. or ( dnase2 ).ti,ab. or ( dnmt3b ).ti,ab. or ( dock2 ).ti,ab. or ( dock8 ).ti,ab. or ( efl1 ).ti,ab. or ( elane ).ti,ab. or ( epg5 ).ti,ab. or ( erbb2ip ).ti,ab. or ( erbin ).ti,ab. or ( ercc4 ).ti,ab. or ( ercc6l2 ).ti,ab. or ( extl3 ).ti,ab. or ( faap24 ).ti,ab. or ( fadd ).ti,ab. or ( fanca ).ti,ab. or ( fancb ).ti,ab. or ( fancc ).ti,ab. or ( fancd2 ).ti,ab. or ( fance ).ti,ab. or ( fancf ).ti,ab. or ( fancg ).ti,ab. or ( fanci ).ti,ab. or ( fancl ).ti,ab. or ( fancm ).ti,ab. or ( fas ).ti,ab. or ( faslg ).ti,ab. or ( fat4 ).ti,ab. or ( fcgr3a ).ti,ab. or ( fcho1 ).ti,ab. or ( fcn3 ).ti,ab. or ( fermt1 ).ti,ab. or ( fermt3 ).ti,ab. or ( foxn1 ).ti,ab. or ( foxp3 ).ti,ab. or ( fpr1 ).ti,ab. or ( g6pc3 ).ti,ab. or ( g6pd ).ti,ab. or ( g6pt1 ).ti,ab. or ( gata2 ).ti,ab. or ( gcs1 ).ti,ab. or ( gfi1 ).ti,ab. or ( gins1 ).ti,ab. or ( havcr2 ).ti,ab. or ( hax1 ).ti,ab. or ( hells ).ti,ab. or ( hmox ).ti,ab. or ( hmox1 ).ti,ab. or ( hoil1 ).ti,ab. or ( hoip ).ti,ab. or ( hyou1 ).ti,ab. or ( icos ).ti,ab. or ( icoslg ).ti,ab. or ( ifih1 ).ti,ab. or ( ifnar1 ).ti,ab. or ( ifnar2 ).ti,ab. or ( ifngr1 ).ti,ab. or ( ifngr2 ).ti,ab. or ( ighm ).ti,ab. or ( igkc ).ti,ab. or ( igll1 ).ti,ab. or ( ikba ).ti,ab. or ( ikbkb ).ti,ab. or ( ikbkg ).ti,ab. or ( ikzf1 ).ti,ab. or ( il10 ).ti,ab. or ( il10ra ).ti,ab. or ( il10rb ).ti,ab. or ( il12b ).ti,ab. or ( il12rb1 ).ti,ab. or ( il12rb2 ).ti,ab. or ( il17f ).ti,ab. or ( il17ra ).ti,ab. or ( il17rc ).ti,ab. or ( il18bp ).ti,ab. or ( il1rn ).ti,ab. or ( il21 ).ti,ab. or ( il21r ).ti,ab. or ( il23r ).ti,ab. or ( il2ra ).ti,ab. or ( il2rb ).ti,ab. or ( il2rg ).ti,ab. or ( il36rn ).ti,ab. or ( il6r ).ti,ab. or ( il6st ).ti,ab. or ( il7r ).ti,ab. or ( ino80 ).ti,ab. or ( irak1 ).ti,ab. or ( irak4 ).ti,ab. or ( irf2bp2 ).ti,ab. or ( irf3 ).ti,ab. or ( irf4 ).ti,ab. or ( irf7 ).ti,ab. or ( irf8 ).ti,ab. or ( irf9 ).ti,ab. or ( isg15 ).ti,ab. or ( itch ).ti,ab. or ( itgb2 ).ti,ab. or ( itk ).ti,ab. or ( jagn1 ).ti,ab. or ( jak1 ).ti,ab. or ( jak3 ).ti,ab. or ( kdm6a ).ti,ab. or ( kmt2a ).ti,ab. or ( kmt2d ).ti,ab. or ( lamtor2 ).ti,ab. or ( lat ).ti,ab. or ( lck ).ti,ab. or ( lig1 ).ti,ab. or ( lig4 ).ti,ab. or ( lpin2 ).ti,ab. or ( lrba ).ti,ab. or ( lyst ).ti,ab. or ( mad2l2 ).ti,ab. or ( magt1 ).ti,ab. or ( malt1 ).ti,ab. or ( map3k14 ).ti,ab. or ( masp2 ).ti,ab. or ( mcm4 ).ti,ab. or ( mefv ).ti,ab. or ( mkl1 ).ti,ab. or ( mll2 ).ti,ab. or ( mogs ).ti,ab. or ( mrtfa ).ti,ab. or ( ms4a1 ).ti,ab. or ( msh6 ).ti,ab. or ( msn ).ti,ab. or ( mthfd1 ).ti,ab. or ( mvk ).ti,ab. or ( myd88 ).ti,ab. or ( mysm1 ).ti,ab. or ( nalp3 ).ti,ab. or ( nbas ).ti,ab. or ( nbn ).ti,ab. or ( nbs1 ).ti,ab. or ( ncf1 ).ti,ab. or ( ncf2 ).ti,ab. or ( ncf4 ).ti,ab. or ( ncstn ).ti,ab. or ( nemo ).ti,ab. or ( nfat5 ).ti,ab. or ( nfe2l2 ).ti,ab. or ( nfkb1 ).ti,ab. or ( nfkb2 ).ti,ab. or ( nfkbia ).ti,ab. or ( nhej1 ).ti,ab. or ( nhp2 ).ti,ab. or ( nlrc4 ).ti,ab. or ( nlrp1 ).ti,ab. or ( nlrp12 ).ti,ab. or ( nlrp3 ).ti,ab. or ( nod2 ).ti,ab. or ( nola2 ).ti,ab. or ( nola3 ).ti,ab. or ( nop10 ).ti,ab. or ( nsmce3 ).ti,ab. or ( oas1 ).ti,ab. or ( orai1 ).ti,ab. or ( ostm1 ).ti,ab. or ( otulin ).ti,ab. or ( palb2 ).ti,ab. or ( parn ).ti,ab. or ( pepd ).ti,ab. or ( pgm3 ).ti,ab. or ( pik3cd ).ti,ab. or ( pik3r1 ).ti,ab. or ( plcg2 ).ti,ab. or ( plekhm1 ).ti,ab. or ( pms2 ).ti,ab. or ( pnp ).ti,ab. or ( pola1 ).ti,ab. or ( pold1 ).ti,ab. or ( pold2 ).ti,ab. or ( pole ).ti,ab. or ( pole1 ).ti,ab. or ( pole2 ).ti,ab. or ( polr3a ).ti,ab. or ( polr3c ).ti,ab. or ( polr3f ).ti,ab. or ( prf1 ).ti,ab. or ( prkcd ).ti,ab. or ( prkdc ).ti,ab. or ( psen ).ti,ab. or ( psen1 ).ti,ab. or ( psenen ).ti,ab. or ( psmb8 ).ti,ab. or ( psmg2 ).ti,ab. or ( pstpip1 ).ti,ab. or ( pten ).ti,ab. or ( ptprc ).ti,ab. or ( pypaf1 ).ti,ab. or ( rab27a ).ti,ab. or ( rac2 ).ti,ab. or ( rad51 ).ti,ab. or ( rad51c ).ti,ab. or ( rag1 ).ti,ab. or ( rag2 ).ti,ab. or ( ranbp2 ).ti,ab. or ( rasgrp1 ).ti,ab. or ( rbck1 ).ti,ab. or ( recql3 ).ti,ab. or ( rel ).ti,ab. or ( rela ).ti,ab. or ( relb ).ti,ab. or ( rfwd3 ).ti,ab. or ( rfx5 ).ti,ab. or ( rfxank ).ti,ab. or ( rfxap ).ti,ab. or ( rhoh ).ti,ab. or ( ripk1 ).ti,ab. or ( rltpr ).ti,ab. or ( rmrp ).ti,ab. or ( rnaseh2a ).ti,ab. or ( rnaseh2b ).ti,ab. or ( rnaseh2c ).ti,ab. or ( rnf168 ).ti,ab. or ( rnf31 ).ti,ab. or ( rnu4atac ).ti,ab. or ( rorc ).ti,ab. or ( rpsa ).ti,ab. or ( rtel1 ).ti,ab. or ( samd9 ).ti,ab. or ( samd9l ).ti,ab. or (samhd1 ).ti,ab. or ( sbds ).ti,ab. or ( sec61a1 ).ti,ab. or ( sema3e ).ti,ab. or ( serping1 ).ti,ab. or ( sh2d1a ).ti,ab. or ( sh3bp2 ).ti,ab. or ( sh3kbp1 ).ti,ab. or ( skiv2l ).ti,ab. or ( slc29a3 ).ti,ab. or ( slc35c1 ).ti,ab. or ( slc37a4 ).ti,ab. or ( slc39a7 ).ti,ab. or ( slc46a1 ).ti,ab. or ( slc7a7 ).ti,ab. or ( slx4 ).ti,ab. or ( smarcal1 ).ti,ab. or ( smarcd2 ).ti,ab. or ( snx10 ).ti,ab. or ( sp110 ).ti,ab. or ( spink5 ).ti,ab. or ( sppl2a ).ti,ab. or ( srp54 ).ti,ab. or ( srp72 ).ti,ab. or ( stat1 ).ti,ab. or ( stat2 ).ti,ab. or ( stat3 ).ti,ab. or ( stat5b ).ti,ab. or ( stim1 ).ti,ab. or ( sting1 ).ti,ab. or ( stk4 ).ti,ab. or ( stn1 ).ti,ab. or ( stx11 ).ti,ab. or ( stxbp2 ).ti,ab. or ( taci ).ti,ab. or ( tap1 ).ti,ab. or ( tap2 ).ti,ab. or ( tapbp ).ti,ab. or ( taz ).ti,ab. or ( tbk1 ).ti,ab. or ( tbx1 ).ti,ab. or ( tcf3 ).ti,ab. or ( tcirg1 ).ti,ab. or ( tcn2 ).ti,ab. or ( terc ).ti,ab. or ( tert ).ti,ab. or ( tfrc ).ti,ab. or ( tgfb1 ).ti,ab. or ( tgfbr1 ).ti,ab. or ( tgfbr2 ).ti,ab. or ( thbd ).ti,ab. or ( ticam1 ).ti,ab. or ( tinf2 ).ti,ab. or ( tirap ).ti,ab. or ( tlr3 ).ti,ab. or ( tmc6 ).ti,ab. or ( tmc8 ).ti,ab. or ( tmem173 ).ti,ab. or ( tnfaip3 ).ti,ab. or ( tnfrsf11a ).ti,ab. or ( tnfrsf13b ).ti,ab. or ( tnfrsf13c ).ti,ab. or ( tnfrsf1a ).ti,ab. or ( tnfrsf4 ).ti,ab. or ( tnfrsf5 ).ti,ab. or ( tnfrsf6 ).ti,ab. or ( tnfrsf9 ).ti,ab. or ( tnfsf11 ).ti,ab. or ( tnfsf12 ).ti,ab. or ( tnfsf5 ).ti,ab. or ( tnfsf6 ).ti,ab. or ( tnfsf7 ).ti,ab. or ( top2b ).ti,ab. or ( tp53 ).ti,ab. or ( tpp1 ).ti,ab. or ( tpp2 ).ti,ab. or ( trac ).ti,ab. or ( traf3 ).ti,ab. or ( traf3ip2 ).ti,ab. or ( trex1 ).ti,ab. or ( trim22 ).ti,ab. or ( trnt1 ).ti,ab. or ( ttc37 ).ti,ab. or ( ttc7a ).ti,ab. or ( tyk2 ).ti,ab. or ( ube2t ).ti,ab. or ( unc13d ).ti,ab. or ( unc93b1 ).ti,ab. or ( ung ).ti,ab. or ( usb1 ).ti,ab. or ( usp18 ).ti,ab. or ( vps13b ).ti,ab. or ( vps45 ).ti,ab. or ( wasp ).ti,ab. or ( wdr1 ).ti,ab. or ( wipf1 ).ti,ab. or ( 'wiskott aldrich syndrome' ).ti,ab. or ( wrap53 ).ti,ab. or ( xiap ).ti,ab. or ( xrcc2 ).ti,ab. or ( xrcc9 ).ti,ab. or ( zap70 ).ti,ab. or ( zbtb24 ).ti,ab. or ( znf341 ).ti,ab. or ( pid ).ti,ab.) 22q11.2 changed to 22q112 (because ‘.’ = syntax error)

**Database: PubMed**

Search from inception until February 3^rd^, 2021. Limits applied: humans, case reports, clinical trial, controlled trial, letter, randomized controlled trial.

Total number of records found: 827

Search Algorithm:

('primary immune regulatory disorders' OR 'inborn errors of immunity' OR 'Primary Immunodeficiency Diseases'[MeSH Terms] OR 'Primary immunodeficiency' OR 'Genetic immunodeficiency') NOT HIV AND (granuloma[Title/Abstract] OR autoimmunity[Title/Abstract] OR autoimmune[Title/Abstract] OR autoinflammation[Title/Abstract] OR 'recurring fever'[Title/Abstract] OR 'periodic fever'[Title/Abstract] OR 'chronic inflammation'[Title/Abstract] OR eczema[Title/Abstract] OR rash[Title/Abstract] OR lymphoproliferation[Title/Abstract] OR lymphadenopathy[Title/Abstract] OR splenomegaly[Title/Abstract] OR hepatosplenomegaly[Title/Abstract] OR 'inflammatory bowel disease'[Title/Abstract] OR enteropathy[Title/Abstract] OR 'chronic diarrhea'[Title/Abstract] OR 'intestinal disease'[Title/Abstract] OR allergy[Title/Abstract]) AND (10p13-p14[Title/Abstract] OR 14q32[Title/Abstract] OR 22q11.2[Title/Abstract] OR acd[Title/Abstract] OR acp5[Title/Abstract] OR actb[Title/Abstract] OR ada[Title/Abstract] OR ada2[Title/Abstract] OR adam17[Title/Abstract] OR adar[Title/Abstract] OR adar1[Title/Abstract] OR aicda[Title/Abstract] OR aire[Title/Abstract] OR ak2[Title/Abstract] OR alpi[Title/Abstract] OR ap1s3[Title/Abstract] OR ap3b1[Title/Abstract] OR ap3d1[Title/Abstract] OR apol1[Title/Abstract] OR arhgef1[Title/Abstract] OR arpc1b[Title/Abstract] OR atm[Title/Abstract] OR atp6ap1[Title/Abstract] OR b2m[Title/Abstract] OR bach2[Title/Abstract] OR baffr[Title/Abstract] OR 'baff-r'[Title/Abstract] OR bcl10[Title/Abstract] OR bcl11b[Title/Abstract] OR blm[Title/Abstract] OR blnk[Title/Abstract] OR brca1[Title/Abstract] OR brca2[Title/Abstract] OR brip1[Title/Abstract] OR btk[Title/Abstract] OR c1q[Title/Abstract] OR c1qa[Title/Abstract] OR c1qb[Title/Abstract] OR c1qc[Title/Abstract] OR c1r[Title/Abstract] OR c1s[Title/Abstract] OR c2[Title/Abstract] OR c2bp1[Title/Abstract] OR c3[Title/Abstract] OR c4[Title/Abstract] OR c4a[Title/Abstract] OR 'c4a+c4b'[Title/Abstract] OR c4b[Title/Abstract] OR c5[Title/Abstract] OR c6[Title/Abstract] OR c7[Title/Abstract] OR c8a[Title/Abstract] OR c8b[Title/Abstract] OR c8g[Title/Abstract] OR c9[Title/Abstract] OR card11[Title/Abstract] OR card14[Title/Abstract] OR card15[Title/Abstract] OR card9[Title/Abstract] OR carmil2[Title/Abstract] OR casp10[Title/Abstract] OR casp8[Title/Abstract] OR ccbe1[Title/Abstract] OR cd19[Title/Abstract] OR cd20[Title/Abstract] OR cd21[Title/Abstract] OR cd247[Title/Abstract] OR cd27[Title/Abstract] OR cd3d[Title/Abstract] OR cd3e[Title/Abstract] OR cd3g[Title/Abstract] OR cd3z[Title/Abstract] OR cd40[Title/Abstract] OR cd40lg[Title/Abstract] OR cd46[Title/Abstract] OR cd55[Title/Abstract] OR cd59[Title/Abstract] OR cd70[Title/Abstract] OR cd79a[Title/Abstract] OR cd79b[Title/Abstract] OR cd81[Title/Abstract] OR cd8a[Title/Abstract] OR cdca7[Title/Abstract] OR cebpe[Title/Abstract] OR cecr1[Title/Abstract] OR cfb[Title/Abstract] OR cfd[Title/Abstract] OR cfh[Title/Abstract] OR cfhr[Title/Abstract] OR cfhr1[Title/Abstract] OR cfhr2[Title/Abstract] OR cfhr3[Title/Abstract] OR cfhr4[Title/Abstract] OR cfhr5[Title/Abstract] OR cfi[Title/Abstract] OR cfp[Title/Abstract] OR cftr[Title/Abstract] OR chd7[Title/Abstract] OR cias1[Title/Abstract] OR cib1[Title/Abstract] OR ciita[Title/Abstract] OR clcn7[Title/Abstract] OR clpb[Title/Abstract] OR copa[Title/Abstract] OR coro1a[Title/Abstract] OR cr2[Title/Abstract] OR csf2ra[Title/Abstract] OR csf2rb[Title/Abstract] OR csf3r[Title/Abstract] OR ctc1[Title/Abstract] OR ctla4[Title/Abstract] OR ctps1[Title/Abstract] OR ctsc[Title/Abstract] OR cxcr4[Title/Abstract] OR cyba[Title/Abstract] OR cybb[Title/Abstract] OR cybc1[Title/Abstract] OR dbr1[Title/Abstract] OR dclre1c[Title/Abstract] OR def6[Title/Abstract] OR dkc1[Title/Abstract] OR dnajc21[Title/Abstract] OR dnase1l3[Title/Abstract] OR dnase2[Title/Abstract] OR dnmt3b[Title/Abstract] OR dock2[Title/Abstract] OR dock8[Title/Abstract] OR efl1[Title/Abstract] OR elane[Title/Abstract] OR epg5[Title/Abstract] OR erbb2ip[Title/Abstract] OR erbin[Title/Abstract] OR ercc4[Title/Abstract] OR ercc6l2[Title/Abstract] OR extl3[Title/Abstract] OR faap24[Title/Abstract] OR fadd[Title/Abstract] OR fanca[Title/Abstract] OR fancb[Title/Abstract] OR fancc[Title/Abstract] OR fancd2[Title/Abstract] OR fance[Title/Abstract] OR fancf[Title/Abstract] OR fancg[Title/Abstract] OR fanci[Title/Abstract] OR fancl[Title/Abstract] OR fancm[Title/Abstract] OR fas[Title/Abstract] OR faslg[Title/Abstract] OR fat4[Title/Abstract] OR fcgr3a[Title/Abstract] OR fcho1[Title/Abstract] OR fcn3[Title/Abstract] OR fermt1[Title/Abstract] OR fermt3[Title/Abstract] OR foxn1[Title/Abstract] OR foxp3[Title/Abstract] OR fpr1[Title/Abstract] OR g6pc3[Title/Abstract] OR g6pd[Title/Abstract] OR g6pt1[Title/Abstract] OR gata2[Title/Abstract] OR gcs1[Title/Abstract] OR gfi1[Title/Abstract] OR gins1[Title/Abstract] OR havcr2[Title/Abstract] OR hax1[Title/Abstract] OR hells[Title/Abstract] OR hmox[Title/Abstract] OR hmox1[Title/Abstract] OR hoil1[Title/Abstract] OR hoip[Title/Abstract] OR hyou1[Title/Abstract] OR icos[Title/Abstract] OR icoslg[Title/Abstract] OR ifih1[Title/Abstract] OR ifnar1[Title/Abstract] OR ifnar2[Title/Abstract] OR ifngr1[Title/Abstract] OR ifngr2[Title/Abstract] OR ighm[Title/Abstract] OR igkc[Title/Abstract] OR igll1[Title/Abstract] OR ikba[Title/Abstract] OR ikbkb[Title/Abstract] OR ikbkg[Title/Abstract] OR ikzf1[Title/Abstract] OR il10[Title/Abstract] OR il10ra[Title/Abstract] OR il10rb[Title/Abstract] OR il12b[Title/Abstract] OR il12rb1[Title/Abstract] OR il12rb2[Title/Abstract] OR il17f[Title/Abstract] OR il17ra[Title/Abstract] OR il17rc[Title/Abstract] OR il18bp[Title/Abstract] OR il1rn[Title/Abstract] OR il21[Title/Abstract] OR il21r[Title/Abstract] OR il23r[Title/Abstract] OR il2ra[Title/Abstract] OR il2rb[Title/Abstract] OR il2rg[Title/Abstract] OR il36rn[Title/Abstract] OR il6r[Title/Abstract] OR il6st[Title/Abstract] OR il7r[Title/Abstract] OR ino80[Title/Abstract] OR irak1[Title/Abstract] OR irak4[Title/Abstract] OR irf2bp2[Title/Abstract] OR irf3[Title/Abstract] OR irf4[Title/Abstract] OR irf7[Title/Abstract] OR irf8[Title/Abstract] OR irf9[Title/Abstract] OR isg15[Title/Abstract] OR itch[Title/Abstract] OR itgb2[Title/Abstract] OR itk[Title/Abstract] OR jagn1[Title/Abstract] OR jak1[Title/Abstract] OR jak3[Title/Abstract] OR kdm6a[Title/Abstract] OR kmt2a[Title/Abstract] OR kmt2d[Title/Abstract] OR lamtor2[Title/Abstract] OR lat[Title/Abstract] OR lck[Title/Abstract] OR lig1[Title/Abstract] OR lig4[Title/Abstract] OR lpin2[Title/Abstract] OR lrba[Title/Abstract] OR lyst[Title/Abstract] OR mad2l2[Title/Abstract] OR magt1[Title/Abstract] OR malt1[Title/Abstract] OR map3k14[Title/Abstract] OR masp2[Title/Abstract] OR mcm4[Title/Abstract] OR mefv[Title/Abstract] OR mkl1[Title/Abstract] OR mll2[Title/Abstract] OR mogs[Title/Abstract] OR mrtfa[Title/Abstract] OR ms4a1[Title/Abstract] OR msh6[Title/Abstract] OR msn[Title/Abstract] OR mthfd1[Title/Abstract] OR mvk[Title/Abstract] OR myd88[Title/Abstract] OR mysm1[Title/Abstract] OR nalp3[Title/Abstract] OR nbas[Title/Abstract] OR nbn[Title/Abstract] OR nbs1[Title/Abstract] OR ncf1[Title/Abstract] OR ncf2[Title/Abstract] OR ncf4[Title/Abstract] OR ncstn[Title/Abstract] OR nemo[Title/Abstract] OR nfat5[Title/Abstract] OR nfe2l2[Title/Abstract] OR nfkb1[Title/Abstract] OR nfkb2[Title/Abstract] OR nfkbia[Title/Abstract] OR nhej1[Title/Abstract] OR nhp2[Title/Abstract] OR nlrc4[Title/Abstract] OR nlrp1[Title/Abstract] OR nlrp12[Title/Abstract] OR nlrp3[Title/Abstract] OR nod2[Title/Abstract] OR nola2[Title/Abstract] OR nola3[Title/Abstract] OR nop10[Title/Abstract] OR nsmce3[Title/Abstract] OR oas1[Title/Abstract] OR orai1[Title/Abstract] OR ostm1[Title/Abstract] OR otulin[Title/Abstract] OR palb2[Title/Abstract] OR parn[Title/Abstract] OR pepd[Title/Abstract] OR pgm3[Title/Abstract] OR pik3cd[Title/Abstract] OR pik3r1[Title/Abstract] OR plcg2[Title/Abstract] OR plekhm1[Title/Abstract] OR pms2[Title/Abstract] OR pnp[Title/Abstract] OR pola1[Title/Abstract] OR pold1[Title/Abstract] OR pold2[Title/Abstract] OR pole[Title/Abstract] OR pole1[Title/Abstract] OR pole2[Title/Abstract] OR polr3a[Title/Abstract] OR polr3c[Title/Abstract] OR polr3f[Title/Abstract] OR prf1[Title/Abstract] OR prkcd[Title/Abstract] OR prkdc[Title/Abstract] OR psen[Title/Abstract] OR psen1[Title/Abstract] OR psenen[Title/Abstract] OR psmb8[Title/Abstract] OR psmg2[Title/Abstract] OR pstpip1[Title/Abstract] OR pten[Title/Abstract] OR ptprc[Title/Abstract] OR pypaf1[Title/Abstract] OR rab27a[Title/Abstract] OR rac2[Title/Abstract] OR rad51[Title/Abstract] OR rad51c[Title/Abstract] OR rag1[Title/Abstract] OR rag2[Title/Abstract] OR ranbp2[Title/Abstract] OR rasgrp1[Title/Abstract] OR rbck1[Title/Abstract] OR recql3[Title/Abstract] OR rel[Title/Abstract] OR rela[Title/Abstract] OR relb[Title/Abstract] OR rfwd3[Title/Abstract] OR rfx5[Title/Abstract] OR rfxank[Title/Abstract] OR rfxap[Title/Abstract] OR rhoh[Title/Abstract] OR ripk1[Title/Abstract] OR rltpr[Title/Abstract] OR rmrp[Title/Abstract] OR rnaseh2a[Title/Abstract] OR rnaseh2b[Title/Abstract] OR rnaseh2c[Title/Abstract] OR rnf168[Title/Abstract] OR rnf31[Title/Abstract] OR rnu4atac[Title/Abstract] OR rorc[Title/Abstract] OR rpsa[Title/Abstract] OR rtel1[Title/Abstract] OR samd9[Title/Abstract] OR samd9l[Title/Abstract] OR samhd1[Title/Abstract] OR sbds[Title/Abstract] OR sec61a1[Title/Abstract] OR sema3e[Title/Abstract] OR serping1[Title/Abstract] OR sh2d1a[Title/Abstract] OR sh3bp2[Title/Abstract] OR sh3kbp1[Title/Abstract] OR skiv2l[Title/Abstract] OR slc29a3[Title/Abstract] OR slc35c1[Title/Abstract] OR slc37a4[Title/Abstract] OR slc39a7[Title/Abstract] OR slc46a1[Title/Abstract] OR slc7a7[Title/Abstract] OR slx4[Title/Abstract] OR smarcal1[Title/Abstract] OR smarcd2[Title/Abstract] OR snx10[Title/Abstract] OR sp110[Title/Abstract] OR spink5[Title/Abstract] OR sppl2a[Title/Abstract] OR srp54[Title/Abstract] OR srp72[Title/Abstract] OR stat1[Title/Abstract] OR stat2[Title/Abstract] OR stat3[Title/Abstract] OR stat5b[Title/Abstract] OR stim1[Title/Abstract] OR sting1[Title/Abstract] OR stk4[Title/Abstract] OR stn1[Title/Abstract] OR stx11[Title/Abstract] OR stxbp2[Title/Abstract] OR taci[Title/Abstract] OR tap1[Title/Abstract] OR tap2[Title/Abstract] OR tapbp[Title/Abstract] OR taz[Title/Abstract] OR tbk1[Title/Abstract] OR tbx1[Title/Abstract] OR tcf3[Title/Abstract] OR tcirg1[Title/Abstract] OR tcn2[Title/Abstract] OR terc[Title/Abstract] OR tert[Title/Abstract] OR tfrc[Title/Abstract] OR tgfb1[Title/Abstract] OR tgfbr1[Title/Abstract] OR tgfbr2[Title/Abstract] OR thbd[Title/Abstract] OR ticam1[Title/Abstract] OR tinf2[Title/Abstract] OR tirap[Title/Abstract] OR tlr3[Title/Abstract] OR tmc6[Title/Abstract] OR tmc8[Title/Abstract] OR tmem173[Title/Abstract] OR tnfaip3[Title/Abstract] OR tnfrsf11a[Title/Abstract] OR tnfrsf13b[Title/Abstract] OR tnfrsf13c[Title/Abstract] OR tnfrsf1a[Title/Abstract] OR tnfrsf4[Title/Abstract] OR tnfrsf5[Title/Abstract] OR tnfrsf6[Title/Abstract] OR tnfrsf9[Title/Abstract] OR tnfsf11[Title/Abstract] OR tnfsf12[Title/Abstract] OR tnfsf5[Title/Abstract] OR tnfsf6[Title/Abstract] OR tnfsf7[Title/Abstract] OR top2b[Title/Abstract] OR tp53[Title/Abstract] OR tpp1[Title/Abstract] OR tpp2[Title/Abstract] OR trac[Title/Abstract] OR traf3[Title/Abstract] OR traf3ip2[Title/Abstract] OR trex1[Title/Abstract] OR trim22[Title/Abstract] OR trnt1[Title/Abstract] OR ttc37[Title/Abstract] OR ttc7a[Title/Abstract] OR tyk2[Title/Abstract] OR ube2t[Title/Abstract] OR unc13d[Title/Abstract] OR unc93b1[Title/Abstract] OR ung[Title/Abstract] OR usb1[Title/Abstract] OR usp18[Title/Abstract] OR vps13b[Title/Abstract] OR vps45[Title/Abstract] OR wasp[Title/Abstract] OR wdr1[Title/Abstract] OR wipf1[Title/Abstract] OR 'wiskott aldrich syndrome'[Title/Abstract] OR wrap53[Title/Abstract] OR xiap[Title/Abstract] OR xrcc2[Title/Abstract] OR xrcc9[Title/Abstract] OR zap70[Title/Abstract] OR zbtb24[Title/Abstract] OR znf341[Title/Abstract] OR pid[Title/Abstract] OR 'DiGeorge Syndrome'[MesH Terms] OR 'Wiskott-Aldrich Syndrome'[MesH Terms] OR 'Autoimmune polyendocrinopathy syndrome, type 1'[MesH Terms] OR 'Ataxia Telangiectasia'[MesH Terms] OR 'Immune Dysregulation, Polyendocrinopathy, Enteropathy, X-Linked Syndrome'[MesH Terms] OR 'Granulomatous Disease, Chronic'[MesH Terms] OR 'Hyper-IgM Immunodeficiency Syndrome, Type 1'[MesH Terms] OR 'Glucosephosphate Dehydrogenase Deficiency'[MesH Terms] OR 'Chediak-Higashi Syndrome'[MesH Terms] OR 'Lymphohistiocytosis, Hemophagocytic'[MesH Terms] OR 'Severe Combined Immunodeficiency'[MesH Terms] OR 'Bruton type agammaglobulinemia'[MesH Terms] OR 'Leukocyte-Adhesion Deficiency Syndrome'[MesH Terms] OR 'Common Variable Immunodeficiency'[MesH Terms])

# List of HPO (Human Phenotype Ontology) umbrella terms and their respective subcategories used for statistical analysis

## Definitions forms of immune dysregulation:

**Granuloma**

- Granulomatosis HP:0002955 and all descendants

- Granuloma HP:0032252 and all descendants

- Renal interstitial granulomas HP:0032641 and all descendants

- Pulmonary granulomatosis HP:0030250 and all descendants

**Autoimmunity**

- Autoimmunity HP:0002960 and all descendants

- Vitiligo HP:0001045 and all descendants

- Alopecia HP:0001596 and all descendants

- Vasculitis HP:0002633 and all descendants

- Type I diabetes mellitus HP:0100651

- Insulin-resistant diabetes mellitus HP:0000831 and all descendants

- Arthritis HP:0001369

- Rheumatoid arthritis HP:0001370 and descendant

- Arthralgia/arthritis HP:0005059

- Migratory arthritis HP:0033037

- Symmetric polyarthritis HP:0040311

- Oligoarthritis HP:0040313

- Autoimmune Hypoparathyroidism HP:0011771

- Coombs-positive hemolytic anemia HP:0004844

- Lupus nephritis HP:0033726

- Interface hepatitis HP:0032220

**Recurrent fever**

- Recurrent fever HP:0001954 and all descendants

- Persistent fever HP:0033399

**Rash**

- Inflammatory abnormality of the skin HP:0011123 and all descendants without „recurrent skin infections“ (and all descendants) and „cutaneous abscess“ (and all descendants)

**Lymphoproliferation**

- Lymphadenopathy HP:0002716 and all descendants

- Splenomegaly HP:0001744 and all descendants

- Hepatomegaly HP:0002240 and all descendants

- Lymphoproliferative disorder HP:0005523

**Gastrointestinal disease**

- Diarrhoea HP:0002014 and all descendants

- Bowel irritability HP:0033628

- Gastrointestinal inflammation HP:0004386 and all descendants

- Colitis HP:0002583 and all descendants

- Abnormal intestine morphology HP:0002242 (Enteropathy)

- Protein-losing enteropathy HP:0002243

- Celiac disease HP:0002608

- Gastrointestinal ulcer HP:0034274 and all descendants

**Allergy**

- Allergy HP:0012393 and all descendants

**Asthma**

- Asthma HP:0002099 and all descendants

**Hemophagocytosis**

- Hemophagocytosis HP:0012156

## Definitions Allergy Categories (only HPO terms appearing in our data are listed):

**Food allergy**

- Food allergy HP:0500093

- Plant product allergy HP:0410338

- Egg allergy HP:0410328

- Plant based food allergy HP:0410332

- Seafood allergy HP:0410333

- Meat allergen allergy HP:0410330

- Cow milk allergy HP:0100327

- Nut food product allergy HP:0410331

**Allergic rhinitis**

- Feather allergy HP:0410326

- Allergic conjunctivitis HP:0007879

- Allergic rhinitis HP:0003193

- Seasonal allergy HP:0012395

- Fungi allergy HP:0410334

- Dust mite allergy HP:0410324

- Animal protein allergy HP:0410320

**Drug allergy**

- Drug allergy HP:0410323

**Other**

- Insect allergy HP:0410335

- Latex allergy HP:0500094

**Unknown**

- Allergy HP:0012393

## Definitions of other allergy-related manifestations (only HPO terms appearing in our data are listed):

**Eczema**

- Eczematoid dermatitis HP:0000964

**Atopic dermatitis**

- Atopic dermatitis HP:0001047

**Urticaria**

- Urticaria HP:0001025

- Cold urticaria HP:0410135

- Chronic idiopathic urticaria HP:0410133

**Anaphylaxis**

- Anaphylactic shock HP:0100845

- Food-induced anaphylaxis HP:0500095

- Drug-induced anaphylaxis HP:0410149

**Eosinophilic esophagitis**

- Eosinophilic infiltration of the esophagus HP:0410151
